# Contribution of supraspinal systems to generation of automatic postural responses

**DOI:** 10.3389/fnint.2014.00076

**Published:** 2014-10-01

**Authors:** Tatiana G. Deliagina, Irina N. Beloozerova, Grigori N. Orlovsky, Pavel V. Zelenin

**Affiliations:** ^1^Department of Neuroscience, Karolinska InstituteStockholm, Sweden; ^2^Barrow Neurological InstitutePhoenix, AZ, USA

**Keywords:** balance control, postural reflexes, reticulospinal neurons, pyramidal tract neurons, rubrospinal neurons, unilateral labyrinthectomy, galvanic vestibular stimulation

## Abstract

Different species maintain a particular body orientation in space due to activity of the closed-loop postural control system. In this review we discuss the role of neurons of descending pathways in operation of this system as revealed in animal models of differing complexity: lower vertebrate (lamprey) and higher vertebrates (rabbit and cat). In the lamprey and quadruped mammals, the role of spinal and supraspinal mechanisms in the control of posture is different. In the lamprey, the system contains one closed-loop mechanism consisting of supraspino-spinal networks. Reticulospinal (RS) neurons play a key role in generation of postural corrections. Due to vestibular input, any deviation from the stabilized body orientation leads to activation of a specific population of RS neurons. Each of the neurons activates a specific motor synergy. Collectively, these neurons evoke the motor output necessary for the postural correction. In contrast to lampreys, postural corrections in quadrupeds are primarily based not on the vestibular input but on the somatosensory input from limb mechanoreceptors. The system contains two closed-loop mechanisms – spinal and spino-supraspinal networks, which supplement each other. Spinal networks receive somatosensory input from the limb signaling postural perturbations, and generate spinal postural limb reflexes. These reflexes are relatively weak, but in intact animals they are enhanced due to both tonic supraspinal drive and phasic supraspinal commands. Recent studies of these supraspinal influences are considered in this review. A hypothesis suggesting common principles of operation of the postural systems stabilizing body orientation in a particular plane in the lamprey and quadrupeds, that is interaction of antagonistic postural reflexes, is discussed.

## INTRODUCTION

Various species from mollusk to man stabilize a particular body orientation in space due to the activity of a feedback postural control system. Any deviation from the desirable body orientation caused by external forces evokes an automatic postural response (corrective movement) aimed at restoration of the initial orientation. Maintenance of a specific body orientation in space (e.g., vertical or dorsal-side-up) is a vital motor function based on inborn neural mechanisms. Numerous studies have been devoted to different aspects of the control of body posture during standing in humans and in some animal models. These studies characterized the motor and EMG patterns of postural reactions, which allowed formulating a number of hypotheses about functional organization of the postural control system (for review see e.g., Horak and Macpherson, 1996; Massion, 1998; Massion et al., 2001; Bouisset and Do, 2008).

During last two decades we have studied the organization and operation of neuronal mechanisms responsible for stabilization of the body orientation in animal models of different complexity – mollusk, lamprey, rabbit, and cat. Comparison of the reactions to similar postural perturbations in evolutionarily remote species revealed some common principles in the organization and operation of their postural mechanisms, as well as some distinctions (Deliagina et al., 2006b). Experiments on simple animal models allow an in depth analysis of the postural neuronal networks, which at present is difficult to perform in higher vertebrates. In this review, we consider mainly the nervous mechanisms responsible for the dorsal-side-up orientation of the animal. Special attention is given to the contribution of supraspinal neuronal mechanisms to the generation of automatic postural responses.

## CONTROL OF BODY ORIENTATION IN LAMPREY

### POSTURAL BEHAVIOR

The lamprey (Cyclostome) is a lower vertebrate animal. The principal organization of its CNS is similar to that in higher vertebrates (Nieuwenhuys and Ten Donkelaar, 1996). This simple animal model presents a unique opportunity for studies of different neuronal mechanisms, including locomotor (see, e.g., Grillner et al., 1991, 1995) and postural networks, which have been analyzed in considerable detail.

**FIGURE 1:**
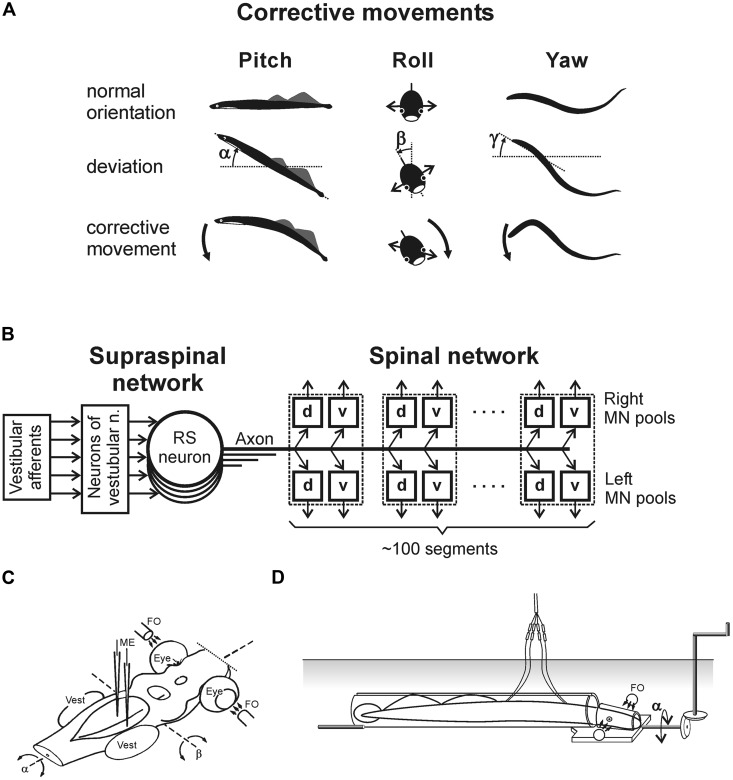
**Experiments on the lamprey. (A)** During regular swimming, the lamprey stabilizes its orientation in the sagittal (pitch) plane, in the transverse (roll) plane, and in the horizontal (yaw) plane. Deviations from the stabilized orientation in these planes (angles α, β, and γ, respectively) evoke corrective motor responses (large arrows) aimed at restoration of the initial orientation. **(B)** Commands for correcting the orientation are formed on the basis of vestibular information, processed by neurons of vestibular nuclei, and transmitted from the brainstem to the spinal cord by axons of reticulospinal (RS) neurons. Motor output of each segment is generated by four motoneuron (MN) pools controlling the dorsal and ventral parts of a myotome on the two sides (*d* and *v* pools). **(C)** Design for *in vitro* experiments. The brainstem was isolated together with vestibular organs (Vest) and eyes. Vestibular stimulation was performed by rotating the preparation around the longitudinal (α) or transverse (β) axes. Visual stimulation was performed by fiber optic (FO). RS neurons (or vestibular afferents) were recorded by microelectrodes (ME). **(D)** Design for *in vivo* experiments. The lamprey was positioned in a narrow tube preventing body movements. Activity of reticulospinal neurons was recorded from their axons in the spinal cord by means of chronically implanted electrodes. Vestibular stimulation was performed by rotation of the setup in the roll plane. Similar setups were used to rotate the animal in the pitch and yaw planes.

The lamprey has two principal behavioral states – a quiescent state when the animal is attached to the substrate with its sucker mouth, and an active state, when it locomotes. The lamprey is capable of several forms of locomotion (Archambault et al., 2001; Islam et al., 2006; Islam and Zelenin, 2008). However, it actively stabilizes the body orientation in space only during the main form of locomotion – fast forward swimming. During this locomotion, orientation of the animal in the sagittal (pitch) and transverse (roll) planes is stabilized in relation to the gravity vector by postural control systems driven by vestibular input (Deliagina et al., 1992a,b; Ullén et al., 1995b; Deliagina and Fagerstedt, 2000; Pavlova and Deliagina, 2002). Vestibular-driven mechanisms also contribute to stabilization of the swimming direction in the horizontal (yaw) plane (Karayannidou et al., 2007). Any deviations from the stabilized body orientation are reflected in vestibular signals, which cause corrective motor responses. In the pitch and yaw planes, these corrective responses occur due to the body bending in the corresponding plane (**Figure 1A**, Pitch and Yaw; Ullén et al., 1995a,b). In the roll plane, the corrections occur due to a change in the direction of locomotor body undulations, from the lateral (left–right) to the oblique one (**Figure 1A**, Roll; Zelenin et al., 2003a).

Usually, the lamprey stabilizes its dorsal-side-up and horizontal body orientation in the transverse and sagittal planes, respectively. However, under certain environmental conditions the stabilized orientation can be changed. For example, asymmetrical illumination of eyes causes a roll tilt of the body toward the more illuminated side (referred as “the dorsal light response”) and this new orientation in the transverse plane is actively stabilized by the animal (Ullén et al., 1995b).

### MAIN COMPONENTS OF POSTURAL CONTROL SYSTEM

**Figure 1B** shows basic components of the postural system in the lamprey. Vestibular afferents (through the neurons of vestibular nuclei) affect reticulospinal (RS) neurons. The RS tract is the main descending pathway in the lamprey (Bussières, 1994), which transmits all commands from the brainstem to the spinal cord, including commands for postural corrections. The majority of RS neurons receiving a specific vestibular input (that is responding to rotation in a definite plane) are active only during fast forward swimming, when the animal actively stabilizes the body orientation in space (Zelenin, 2011). Vestibulospinal pathways in the lamprey are poorly developed, contain small number of fibers, terminate in the rostral spinal segments (Bussières, 1994), and produce very weak effects on the motor output (Zelenin et al., 2003b).

The spinal network is responsible for the transformation of RS commands into the motor pattern of postural corrections. This network includes interneurons, as well as four motoneuron (MN) pools in each segment (**Figure 1B**) that innervate the dorsal and ventral parts of a myotome on the two sides. The spinal mechanisms transforming RS commands into the motor pattern of postural corrections are rather complex. For example, signals from intraspinal stretch receptor neurons monitoring the lamprey’s body configuration can modify the spinal networks decoding these commands. Thus, the effects of RS commands may depend on the phase and amplitude of locomotor body undulations (Hsu et al., 2013).

### SENSORY INPUTS TO NEURONS OF POSTURAL NETWORKS

To analyse operation of the postural networks, the following questions were addressed: (i) how individual vestibular afferents respond to a deviation of the body from the desirable orientation, (ii) how individual RS neurons respond to this vestibular input, (iii) how postural commands transmitted by individual neurons are decoded in the spinal cord, which results in the generation of postural corrections. To answer these questions, a number of animal preparations and experimental techniques have been developed (**Figures 1C,D** and **3A**; Deliagina et al., 1992a,b, 2000a; Orlovsky et al., 1992; Deliagina and Fagerstedt, 2000; Pavlova and Deliagina, 2002; Karayannidou et al., 2007).

**FIGURE 2:**
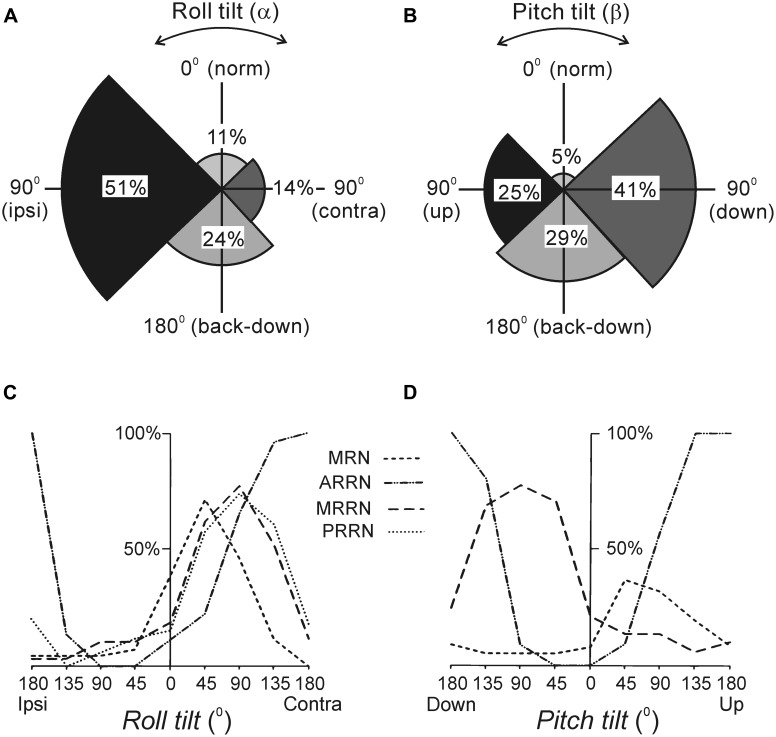
**Reactions of supraspinal network to rotation in the transverse (roll) and sagittal (pitch) planes. (A,B)** Proportion of otolith afferents with different zones of spatial sensitivity in the roll **(A)** and pitch **(B)** planes. Angular zones of sensitivity and percentage of afferents in each zone are indicated. **(C,D)** Summary diagrams of responses to roll and pitch in different reticular nuclei. The relative number of neurons active at different positions is presented as a function of roll **(C)** and pitch **(D)**. For simplicity, neither the group of MRRN neurons sensitive to nose-up pitch tilt nor the groups of PRRN neurons with zones of sensitivity distributed over the whole space are shown in **(D)**. Designations of reticular nuclei: PRRN, posterior rhombencephalic; MRRN, middle rhombencephalic; ARRN, anterior rhombencephalic; MRN, mesencephalic.

As with other vertebrates, the lamprey has canal and otolith afferents (Lowenstein et al., 1968). The canal afferents respond to a change in orientation with a high-frequency burst (Deliagina et al., 1992b). In the transverse plane, they respond to rotation toward ipsi-side down. Pitch tilt revealed two groups of canal afferents responding to rotation toward either nose-up or nose-down. The otolith afferents respond both to a change of position and to a maintained new position. These afferents were classified in several groups according to their zones of sensitivity (**Figures 2A,B**). For roll, the largest group has maximal sensitivity around a 90° tilt to the ipsilateral side (**Figure 2A**). For pitch, there are groups responding with maximal sensitivity at 90° nose-down and 90° nose-up (**Figure 2B**). In addition, a group responding at up-side-down position (180°) was revealed (**Figures 2A,B**). A minority of afferents are active during normal (dorsal-side-up) orientation and during contralateral roll.

Most RS neurons respond to the contralateral roll tilt and have both dynamic and static response components. The zones of spatial sensitivity differ in different reticular nuclei; together they cover the whole range of possible inclinations in the transverse plane (**Figure 2C**). The roll-sensitive RS neurons are driven mainly by excitatory contralateral vestibular input (Deliagina and Pavlova, 2002). They also receive weak input from the ipsilateral labyrinth, which supplements the contralateral one (Deliagina and Pavlova, 2002). In addition, they receive excitatory and inhibitory inputs from the ipsilateral and contralateral eye, respectively, which affect the magnitude of their response to roll (Deliagina et al., 1993; Deliagina and Fagerstedt, 2000).

In the pitch plane, most RS neurons respond either to the nose-up pitch tilt, or to the nose-down pitch tilt (Deliagina et al., 1992a; Orlovsky et al., 1992; Pavlova and Deliagina, 2002). The neurons of these two populations reside in all reticular nuclei, but in different proportions (**Figure 2D**). The RS neurons responding to nose-up pitch tilt are driven mainly by an excitatory input from the contralateral labyrinth. By contrast, nose-down RS neurons receive excitatory inputs from both labyrinths (Pavlova and Deliagina, 2003). About a quarter of RS neurons respond to both roll and pitch tilts suggesting that these neurons are partly shared by the pitch and roll control systems (Pavlova and Deliagina, 2003; Zelenin et al., 2007).

Finally, in the yaw plane, most RS neurons respond to contralateral turn due to an excitatory input mainly from the contralateral labyrinth (Karayannidou et al., 2007).

### ENCODING AND DECODING OF RS POSTURAL COMMANDS

**FIGURE 3:**
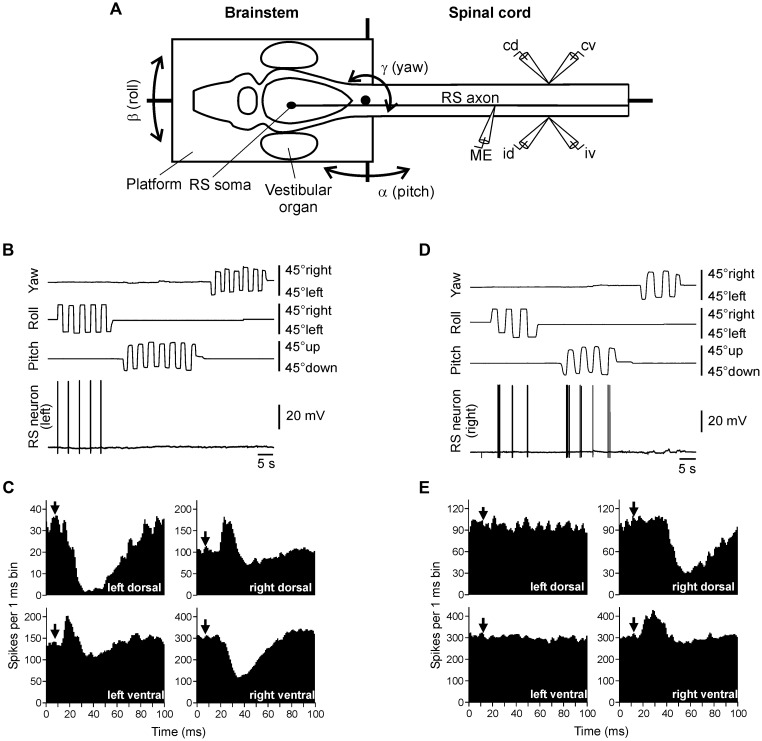
**Vestibular inputs and motor outputs of individual RS neurons. (A)** The brainstem – spinal cord preparation with vestibular organs was used for studying vestibular inputs to individual RS neurons and their motor effects. The preparation was positioned in a chamber and perfused with Ringer solution. The brainstem with vestibular organs could be rotated around three axes: transverse (pitch), longitudinal (roll), and vertical (yaw). D-glutamate was applied to the spinal cord to elicit fictive locomotion. Individual neurons were recorded from their axons in the spinal cord. To stimulate a neuron, positive current pulses were passed through the recording intracellular microelectrode (ME). Activity of MNs was recorded bilaterally in the segment 30 by suction electrodes, from the dorsal and ventral branches of a ventral root (*id*, ipsilateral dorsal branch; *iv*, ipsilateral ventral; *cd*, contralateral dorsal; *cv*, contralateral ventral). **(B,C)** An RS neuron that contributed only to stabilization of the body orientation in the transverse plane. The neuron fired spikes in response to right (contralateral) roll tilts only **(B)**. The neuron evoked excitation in the left (ipsilateral) ventral and right (contralateral) dorsal branches of the ventral roots and inhibition in the right ventral and left dorsal branches** (C)**. **(D,E)** An RS neuron that contributed to stabilization of the body orientation in both transverse and sagittal planes. The neuron fired spikes in response to left (contralateral) roll tilts and nose-up pitch tilts **(D)**. The neuron evoked excitation in the ipsilateral ventral branch of the ventral root and inhibition in the ipsilateral dorsal branch **(E)**. In panels **(C,E)**, a post-RS-spike histogram was generated for the spikes of motoneurons recorded in the dorsal and ventral branches of the left and right ventral roots. The moment of RS spike occurrence at the stimulated site was taken as the origin of the time axis in the histogram. Arrows indicate the time of arrival of the RS spike to segment 30 (where motor output was monitored). Typically, responses to several thousands of RS spikes were used for generation of a histogram.

To characterize the sensory-motor transformation in postural neuronal networks, a special technique was developed to assess both vestibular inputs and motor effects of individual RS neurons (**Figure 3**; Zelenin et al., 2001, 2007).

The motor effects of individual neurons were qualitatively the same along the whole extent of the axon (Zelenin et al., 2001), and thus could be characterized by a combination of influences on the four MN pools in any segment (muscle synergy; **Figure 1B**).

The majority (68%) of RS neurons with specific vestibular inputs and specific motor effects respond to rotation only in one of the three main planes, as the neuron in **Figure 3B**. This neuron fires spikes in response to contralateral roll tilts, and does not respond to rotation in the yaw and pitch planes. Thus, it belongs to the roll control system. Motor effects of this neuron are shown in **Figure 3C**. They include activation of the MN pools projecting to the ipsi-ventral and contra-dorsal myotomes, and inhibition of those projecting to the ipsi-dorsal and contra-ventral myotomes. In the swimming lamprey, this pattern would lead to a change in the direction of locomotor body undulations, from lateral to oblique, resulting in a roll torque directed opposite to the initial turn (**Figure 1A**, Roll; Zelenin et al., 2007). In the majority of RS neurons there is a strong correlation between vestibular inputs and motor effects, as in the neuron shown in **Figure 3B** (Zelenin et al., 2007). Most often, the neuron produced a motor pattern causing a torque, which would oppose the initial rotation that activated the neuron.

About quarter of RS neurons responded to rotation in more than one plane (as the neuron shown in **Figure 3D**). This neuron responded to left (contralateral) roll tilts and to nose-up pitch tilts but did not respond to rotation in the yaw plane. The neuron excited the ipsilateral ventral MNs and inhibited the ipsilateral dorsal MNs (**Figure 3E**), thus contributing to postural corrections caused by the left roll tilt (that is activation of the right ventral and left dorsal myotomes, and inhibition of right dorsal and left ventral myotomes), as well as to the nose-up pitch tilt (that is activation of both ventral myotomes and inhibition of both dorsal myotomes). Most of the neurons responding to rotation in more than one plane produced the motor pattern contributing to postural corrections in the corresponding planes.

**FIGURE 4:**
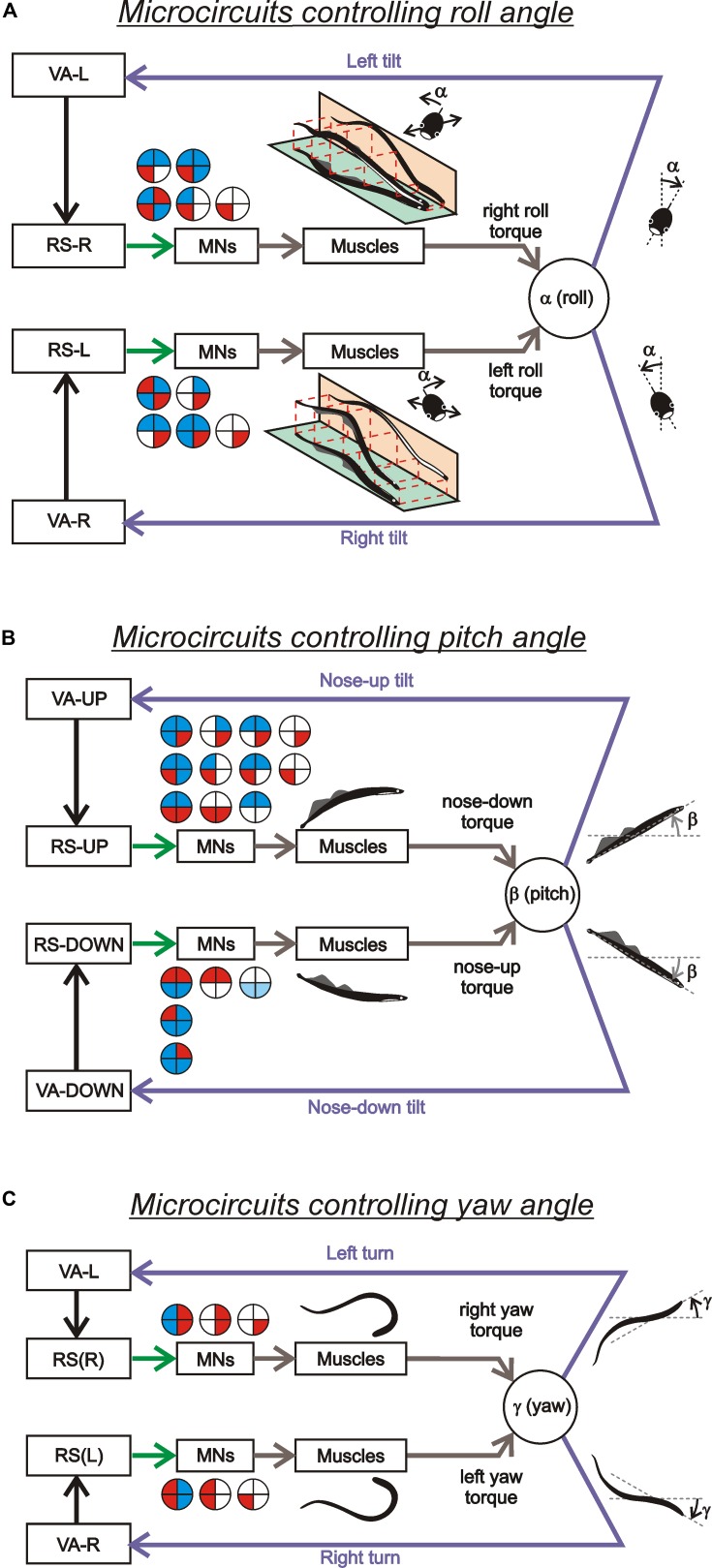
**Sensory-motor transformation in neuronal networks underlying operation of the roll, pitch, and yaw control systems.** Relationships between vestibular responses and motor effects in individual RS neurons of the roll **(A)**, pitch **(B)**, and yaw **(C)** control systems. The neurons were divided into groups [RS-L, RS-R, RS-UP, RS-DOWN, RS(L), and RS(R)] according to their inputs (vestibular responses). For each group, the patterns of motor effects in its neurons are shown as circle diagrams, with the quadrants representing the motoneuronal pools (MNs) projecting to the corresponding parts of the myotomes. Different colors designate the type of effect (excitation – red, inhibition – blue, no effect – white). Each RS neuron evoked a motor pattern (or a part of the pattern) opposing the initial turn that activated the neuron.

Thus, individual RS neurons transform sensory information about the body orientation into motor commands that produce corrections of orientation. The closed-loop microcircuits formed by individual RS neurons belonging to a particular (roll, pitch, or yaw) postural system operate in parallel to generate the resulting motor responses that counteract the postural disturbances (**Figure 4**).

These results support a point of view that each type of postural corrections in humans and quadrupeds is based on a combination of specific muscle synergies (for review, see Ting, 2007). One can suggest that, similar to the lamprey, in other vertebrates these synergies are also activated by specific descending neurons.

### FUNCTIONAL MODEL OF POSTURAL SYSTEM

The aforementioned data allowed to formulate conceptual models of the postural systems responsible for stabilization of the body orientation in the roll, pitch, and yaw planes (Deliagina and Orlovsky, 2002; see also Deliagina and Fagerstedt, 2000; Zelenin et al., 2001; Pavlova and Deliagina, 2002; Karayannidou et al., 2007; Zelenin et al., 2007).

**FIGURE 5:**
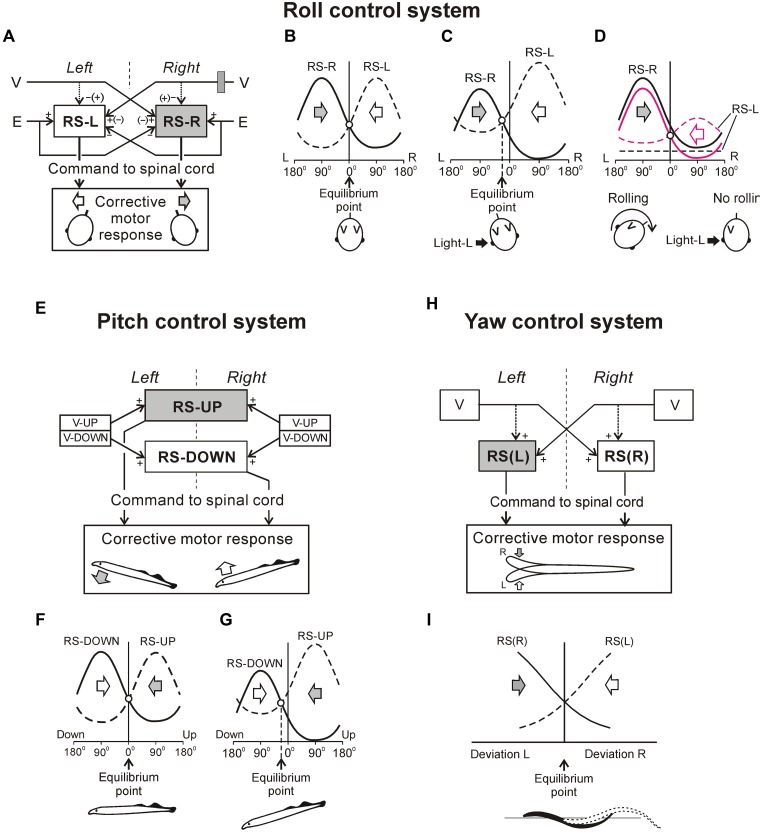
**Conceptual models of systems controlling orientation in different planes. (A–D)** Roll control system.** (A)** Two groups of RS neurons (RS-L and RS-R) receive inputs from the labyrinths (V) and eyes (E); they affect the spinal networks to evoke rolling of the lamprey. The signs (+ and –) indicate the major effects on RS neurons produced by sensory inputs, the signs in brackets – the minor effects. **(B)** Operation of the system when driven only by vestibular inputs. The curves represent activity in RS-R and RS-L as a function of roll angle (L, left tilt; R, right tilt). Vestibular input causes activation of RS-R and RS-L with the contralateral tilt. Direction of rolling caused by RS-R and RS-L is indicated by the gray and white arrows, respectively. The system has an equilibrium point at 0° (dorsal-side-up orientation). **(C)** Operation of the system when the left eye is illuminated. This visual input (a black arrow; Light-L) causes a shift of the equilibrium point to the left and the corresponding tilt of the animal. **(D)** Effect of the right unilateral labyrinthectomy (indicated by gray rectangle in **A**). The RS activity after the right labyrinthectomy is shown by black solid and interrupted lines. The system has no equilibrium point and the animal continuously rolls to the right. Rolling could be abolished by means of left eye illumination causing activation of RS-L and some inactivation of the RS-R (shown by red interrupted and solid lines, respectively) resulting in re-creation of the equilibrium point. **(E–G)** Pitch control system.** (E)** Two groups of RS neurons, RS-UP and RS-DOWN, receive excitatory inputs from vestibular afferents activated by nose-up (V-UP) and nose-down (V-DOWN) pitch tilt, respectively. Each of the RS-UP and RS-DOWN groups sends a command to the spinal cord causing downward and upward turning of the lamprey, respectively, (gray and white arrows). **(F)** Operation of the system during horizontal swimming. Curves represent the activity of RS-UP and RS-DOWN and their motor effects as a function of the pitch angle. Vestibular input causes activation of the groups with upward and downward tilt, respectively. Direction of turning caused by RS-UP and RS-DOWN is indicated by gray and white arrows, respectively. System has an equilibrium point at 0° (horizontal orientation). **(G)** Operation of the system under high water temperature (the activity of RS-UP increased relative to that of RS-DOWN). Equilibrium point is displaced toward the down pitch angles. Insets in **(F,G)** show the stabilized body orientation. **(H,I)** Yaw control system. **(H)** Two groups of RS neurons, RS(R) and RS(L), are driven by vestibular afferents from the left and right vestibular organs (V). As a result of these inputs, RS-R and RS-L respond to the left and right yaw turn, respectively. RS-R and RS-L affect the spinal network and cause right and left corrective lateral turn of the lamprey, respectively, (gray and white arrows). Solid lines indicate the major effects on RS neurons produced by vestibular organs; interrupted lines indicate the minor effects. **(I)** Operation of the system during swimming. Two curves represent the activity of RS-R and RS-L groups caused by a dynamic deviation of the head movement from the rectilinear one. Motor effect of each RS group is proportional to its activity. Direction of turning caused by RS-R and RS-L is indicated by the gray and white arrows, respectively. System has an equilibrium point where the effects of RS-R and RS-L are equal to each other.

The functional model of the roll control system is shown in **Figure 5A**. The key elements of the model are two subgroups of RS neurons, the left (RS-L), and the right (RS-R). Due to vestibular inputs, the activity of RS neurons is orientation-dependent with its peak at approximately 90° of contralateral roll tilt (**Figure 5B**). The two subgroups also receive an excitatory input from the ipsilateral eye and an inhibitory input from the contralateral eye. Each of the subgroups, via spinal mechanisms, elicits ipsilateral rotation of the lamprey (**Figures 5A,B**, the white and black thick arrows). The system stabilizes an orientation with equal activities of RS-L and RS-R. At normal environmental conditions this occurs at the dorsal-side-up orientation of the body in the roll plane (equilibrium point in **Figure 5B**). The stabilized orientation can be changed by adding an asymmetrical bias to RS-L and RS-R activities, for example, through asymmetrical visual inputs to RS neurons. Illumination of an eye causes additional excitation of the ipsilateral RS neurons and inhibition of the contralateral ones; this will result in a shift of the equilibrium point of the system toward the illuminated eye and stabilization of the new tilted orientation (**Figure 5C**). These predicted modifications in RS-L and RS-R activities caused by asymmetrical illumination of eyes were found experimentally (Deliagina and Fagerstedt, 2000). This explains the neural mechanism of the dorsal light response, that is, a roll tilt toward the illuminated eye (**Figure 5C**, inset; Deliagina et al., 1992a, 1993; Ullén et al., 1996).

The model can also explain motor deficits in the lamprey caused by the unilateral labyrinthectomy (UL). It is known that UL severely impairs locomotion and postural control in vertebrates. The main deficit caused by UL in the lamprey is rolling, i.e., continuous rotation of the swimming animal around the longitudinal body axis (Deliagina, 1995, 1997a). As shown in **Figure 5D** by a black interrupted line, due to abolition of the excitatory input from the removed right labyrinth, RS-L neurons become inactivated. As a result, the RS-R and RS-L curves do not intersect, the equilibrium point is absent, and RS-R neurons cause continuous rolling to the right. The rolling can be stopped by rising RS-L activity (red interrupted line) so that the two activity curves intersect again. Activation of RS-L neurons can be done either by asymmetrical visual input (illumination of the left eye), or by continuous electrical stimulation of the right vestibular or left optic nerve (Deliagina, 1997b). The changes in activity of RS-L and RS-R neurons predicted by the model were later demonstrated experimentally (Deliagina and Pavlova, 2002). One of the methods for restoration of equilibrium control after UL (electrical stimulation of the stump of the transected vestibular nerve) developed for the lamprey was successfully tested on the rat (Deliagina et al., 1997), suggesting a similarity of the roll control mechanisms in these evolutionary remote species.

The validity of the functional model of the roll control system under dynamic close-to-normal conditions was tested in experiments with a neuro-mechanical model (Zelenin et al., 2000). The lamprey’s body was attached to a platform, orientation of which was controlled by RS-L and RS-R neurons recorded by implanted electrodes. The system was able to maintain the dorsal-side-up body orientation, as well as to reproduce the effects of UL, of asymmetrical illumination of eyes, etc.

A functional model of the pitch control system is shown in **Figures 5E–G** (Pavlova and Deliagina, 2002). Two antagonistic subgroups of RS neurons, RS-UP and RS-DOWN, are driven by vestibular afferents responding to the nose-up pitch tilt (V-UP) and nose-down pitch tilt (V-DOWN), respectively. Due to these vestibular inputs, the activity of RS-UP and RS-DOWN and their motor effects are orientation-dependent (**Figure 5F**). The RS-UP subgroup causes a downward turn of the lamprey, whereas RS-DOWN causes an upward turn (gray and white arrows in **Figures 5E–G**). The system stabilizes the orientation with equal activities of the RS-UP and RS-DOWN groups. Normally this occurs at the zero pitch angle (the horizontal orientation of the body in the pitch plane, equilibrium point in **Figure 5F**). The stabilized orientation can be changed by adding an asymmetrical bias to RS-UP and RS-DOWN activities. A factor, which presumably causes a downward turn of the animal (higher temperature), affects the vestibular responses in RS-UP and RS-DOWN differently (Pavlova and Deliagina, 2002). This results in an increase in the ratio of RS-UP activity to RS-DOWN activity. Because of the increase in the UP/DOWN ratio, an intersection of the two activity curves is shifted from 0° toward the downward tilt angles (**Figure 5G**). This new pitch angle (equilibrium point) is stabilized by the pitch control system.

**Figures 5H,I** presents a conceptual model of the yaw control system (Karayannidou et al., 2007). Two subgroups of RS neurons (RS-L and RS-R) are driven by vestibular inputs mainly from the contralateral labyrinth (**Figure 5H**), so that they are activated with contralateral yaw turn (**Figure 5I**). When activated, RS-L and RS-R subgroups evoke a corrective yaw turn, that is, rotation opposite to the initial turn. If, for example, an external force turns the lamprey to the left, the RS-R subgroup is activated by vestibular input and elicits a corrective turn of the animal to the right, resulting in restoration of the initial orientation in the yaw plane. Thus the yaw control system counteracts any deviations from the rectilinear swimming caused by external factors.

## MAINTENANCE OF LATERAL STABILITY DURING STANDING IN QUADRUPEDS

Maintenance of lateral stability during standing and locomotion is an important function of the postural system in terrestrial quadrupeds. In this section we consider the neural mechanisms responsible for stabilization of the dorsal-side-up body orientation in the rabbit and cat during standing. We will then compare these mechanisms with the roll control system in the lamprey considered above.

Nervous mechanisms responsible for lateral stability in quadrupeds during locomotion (Matsuyama and Drew, 2000; Karayannidou et al., 2009a; Musienko et al., 2014), or during voluntary movements (Schepens et al., 2008; Yakovenko et al., 2011; Cullen, 2012) are out of the scope of this review.

### POSTURAL REACTIONS ENSURING LATERAL STABILITY IN QUADRUPEDS

**FIGURE 6:**
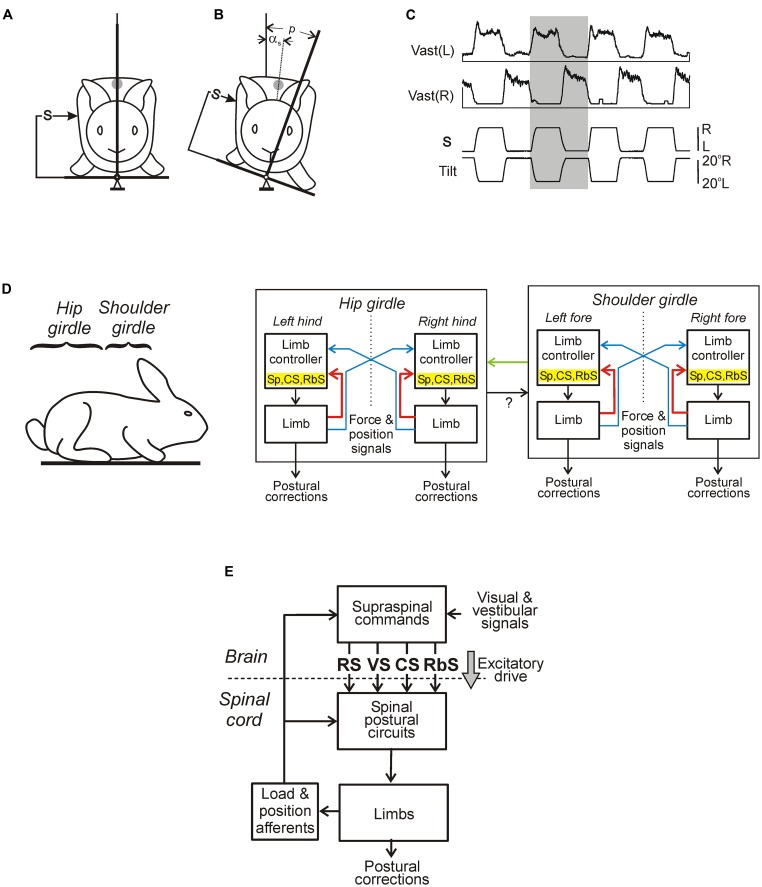
**Maintenance of body orientation in the transverse plane in the standing rabbit. (A,B)** Experimental design for testing postural responses to lateral tilts of the support surface. The platform tilt (p), the trunk tilt after execution of postural correction (α_s_), as well as the position of mechanical sensor (S) measuring the lateral displacement of the trunk in relation to the platform (the trunk corrective movement) are indicated. **(C)** Motor and EMG responses to trapezoidal tilts. Vast(L) and Vast(R) are left and right m. vastus lateralis, respectively. **(D)** Functional model of the postural system stabilizing the trunk orientation in the transverse plane. Lateral stability of the anterior and posterior parts of the body (shoulder and hip girdles) is maintained by two relatively independent sub-systems. Each sub-system contains two controllers (for the right and left limbs) generating a part of corrective limb movement in response to sensory input from the same limb (red lines), spinal postural limb reflexes (Sp), corticospinal (CS), and rubrospinal (RbS) neurons are parts of this mechanism. Another part of corrective limb movement is produced in response to influences from the contralateral limb (blue lines). Coordination between these subsystems is primarily based on influences of the anterior sub-system on the posterior one (green lines). **(E)** Main components of the postural system in quadrupeds. Two closed-loop mechanisms participate in the postural control. Spinal circuits generate postural limb reflexes, and their effects are added to the effects of supraspinal commands, which are generated on the basis of sensory information, and transmitted by the major descending tracts reticulospinal (RS), vestibulospinal (VS), corticospinal (CS), and rubrospinal (RbS). A gray arrow indicates the tonic supraspinal drive that activates the spinal postural circuits.

In standing animals, a lateral tilt of the support surface causes a lateral body sway and evokes a compensatory postural reaction – extension of the limbs on the side moving down and flexion of the limbs on the opposite side. These limb reactions reduce the lateral body sway and move the dorso-ventral trunk axis toward the vertical (**Figures 6A,B**; Deliagina et al., 2000b, 2006a; Beloozerova et al., 2003a). These limb movements are caused by an increase in the limb extensor activity on the side moving down and its decrease in the opposite limb (**Figure 6C**). The somatosensory inputs from the limbs play a major role for elicitation of the postural reactions (Deliagina et al., 2000b; Beloozerova et al., 2003a), except for the case of very high tilt velocity (Macpherson et al., 2007). Usually the system for trunk stabilization operates as a unit, but under certain environmental conditions it dissociates into two relatively independent sub-systems responsible for stabilization of the anterior and posterior parts of the trunk, respectively, (**Figure 6D**). They are driven by somatosensory inputs from the corresponding limbs (Beloozerova et al., 2003a; Deliagina et al., 2006a). Coordination between these sub-systems is primarily based on influences of the anterior sub-system on the posterior one (Deliagina et al., 2006a). It was demonstrated that each sub-system contains two mechanisms – limb controllers for the right and left limbs, generating a part of the corrective limb movement in response to sensory input from the same limb; another part is formed on the basis of sensory influences from the contralateral limb (Deliagina et al., 2006a). Such a functional organization is similar to that of the locomotor system in quadrupeds; it was suggested that a control system consisting of semi-autonomous sub-systems better adapts to complicated environmental conditions (Orlovsky et al., 1999).

Besides postural reactions to lateral tilts, reactions to some other perturbations of balance in the standing cat were investigated, including the reaction to lateral translation of the supporting platform (Macpherson, 1988a,b), to lateral push (Karayannidou et al., 2009a), and to drop of support under one of the limbs (Dufossé et al., 1982; Stapley and Drew, 2009). All these perturbations affect balance in the transverse plane, but it is rapidly compensated due to postural reactions caused by specific muscle synergies. As in the tilt task, these reactions are mainly due to somatosensory input from the limbs. It was reported (Honeycutt et al., 2007, 2008) that input from Group I and II muscle spindle afferents is critically important for directionally appropriate muscle activation in response to horizontal translation of one limb. Thus, in the translation task, the functional organization of the system seems to be similar to that in the tilt task, in which a considerable part of the corrective movement of the limb is generated in response to sensory input from the same limb (**Figure 6D**).

In humans, postural reactions to different perturbations (including lateral tilts and lateral translations of support) have been characterized in a number of studies (e.g., Henry et al., 1998; Carpenter et al., 1999). These data show that the reactions are due to the feedback mechanisms driven, to a large extent, by the somatosensory input from the limbs, similar to quadrupeds. However, in contrast to quadrupeds, vestibular input significantly contributes to their generation (Carpenter et al., 2001).

### MAIN COMPONENTS OF POSTURAL CONTROL SYSTEM

**FIGURE 7:**
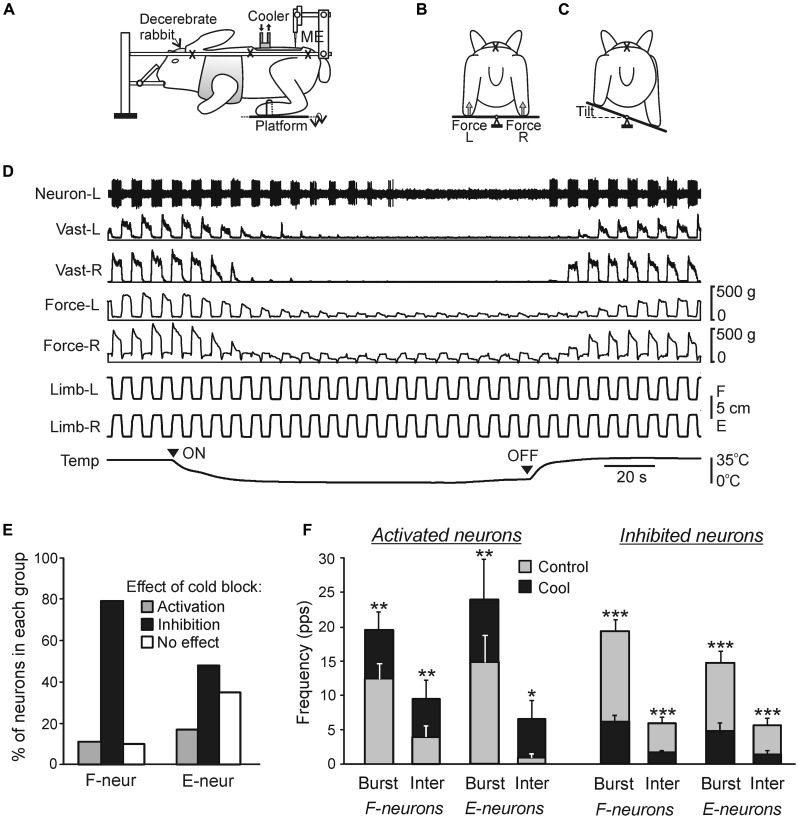
**Effects of reversible spinalization on postural limb reflexes and spinal neurons presumably mediating these reflexes. (A–C)** Details of the experimental design.** (A)** The decerebrate rabbit was fixed in a rigid frame (crosses indicate the fixation points). Activity of spinal neurons from L5 was recorded by a microelectrode (ME). To evoke PLRs, the hindlimbs were positioned on a platform **(B)** periodically tilted in the transverse plane **(B,C)**. The contact forces under the left and right hindlimbs were measured by the force sensors (**B**, Force L and Force R, respectively). **(D)** An example of the effect of reversible spinalization on PLRs and on the activity of a neuron recorded on the left side of spinal segment L5. During the experiment, periodical anti-phase loading/unloading and flexion/extension movements of the left and right limbs were produced by tilting the support platform. The contact force and the EMG of vastus lateralis (Vast) were recorded bilaterally along with the activity of the neuron. Trace *Temp* shows temperature of a cooler placed on the dorsal surface of the spinal cord at T12. Arrowheads ON and OFF indicate the onset of cooling and the onset of re-warming, respectively. Before cooling, tilts of the platform caused PLRs, i.e., activation of extensors during limb flexion/loading and decrease in their activity during limb extension/unloading (left part of recording). Note the disappearance of PLRs (EMG, force), and neuron responses to tilts during cooling, and their re-appearance during re-warming. **(E,F)** Effects of the reversible spinalization on spinal neurons mediating PLRs. **(E)** Proportion of F- and E-neurons activated, inactivated, or unaffected by could block. **(F)** Effect on the mean burst and interburst frequencies of F- and E-neurons activated and inactivated by the cold block. ^∗^*p* < 0.05, ^∗∗^*p* < 0.01, ^∗∗∗^*p* < 0.001, *t*-test.

The main components of the sub-systems maintaining the dorsal-side-up orientation of the trunk are shown in **Figure 6E**. Somatosensory information from the limbs affects the spinal networks directly; it is also sent to the brain where it contributes to formation of supraspinal postural commands transmitted to the spinal cord through different descending pathways. The fact that the premammillary decerebrated rabbit generates postural corrections in response to lateral tilts of the support surface (Musienko et al., 2008) suggests that basic postural networks reside in the brainstem, cerebellum, and spinal cord, and the forebrain contributions are not crucial. However, the value of these corrections is reduced, indicating that input from the forebrain increases excitability of the basic postural networks. An essential part of limb reactions to tilts is postural limb reflexes (PLRs) driven by stretch and load receptors of the limbs; they were studied in the decerebrate rabbit (**Figure 7**; Musienko et al., 2010; Hsu et al., 2012). The EMG pattern of PLRs can be evoked in acute spinal rabbits subjected to the epidural electrical stimulation of the spinal cord (Musienko et al., 2010). This finding suggests that the spinal cord contains the networks generating PLRs, and in intact animals they are activated by the tonic supraspinal drive from the posture-related brain structures (such as the ventral tegmental field and mesencephalic locomotor region; Musienko et al., 2008). However, spinal PLRs are very weak (Mori, 1987; Musienko et al., 2010), suggesting a crucial role of phasic supraspinal commands in the generation of postural corrections.

The conclusion about a crucial role of the brainstem-cerebellum-spinal mechanisms for lateral stability was supported by Honeycutt and colleagues (Honeycutt et al., 2009; Honeycutt and Nichols, 2010) who demonstrated the persistence of essential features of postural reactions to support translation in decerebrate cats.

There are some indirect evidences suggesting that in humans, cortex does not contribute to triggering the initial (short-latency) phase of postural responses to external perturbations (Dietz et al., 1984, 1985; Quintern et al., 1985; Ackermann et al., 1990, 1991; Berger et al., 1990). Thus, it seems likely that in humans (as in terrestrial quadrupeds) the brainstem–cerebellum–spinal cord mechanisms are responsible for the initiation of postural reactions.

Chronic spinal cats can be trained to stand and produce postural reactions to support translation (Fung and Macpherson, 1999). However, the underlying muscle synergies are distorted, the response latencies are longer than normal, and the response amplitude is small (Macpherson and Fung, 1999; Chvatal et al., 2013). This further demonstrates a crucial role of supraspinal phasic commands and tonic drive for normal functioning of the spinal postural networks.

### NEURONS OF SPINAL POSTURAL NETWORKS

Two groups of spinal interneurons (F and E) were found, activity of which strongly correlated with PLRs, suggesting their participation in PLRs generation (Hsu et al., 2012; Zelenin et al., 2013). F-neurons were excited in-phase with extensors of the ipsilateral limb, while E-neurons – in anti-phase (as the neuron in **Figure 7D**). Presumably, at least some F-neurons and E-neurons participate, respectively, in the excitation and inhibition of extensor motoneurons (EMNs) of the ipsilateral limb. The modulation of F- and E-neurons was primarily determined by somatosensory input from the ipsilateral limb. In the framework of the functional model of the postural system stabilizing trunk orientation in the transverse plane (**Figure 6D**) these neurons belong to the feedback mechanism generating corrective limb movements on the basis of sensory information from the same limb.

The recently developed method of “reversible spinalization” (a temporary cold block of the signal transmission in spinal pathways) allowed studying the contribution of supraspinal influences to the activity of individual F- and E-neurons (**Figure 7**; Zelenin et al., 2013). Elimination of supraspinal commands produced diverse but mostly inhibitory effects on F- and E-neurons (**Figure 7E**). A small proportion of neurons was activated during cooling, suggesting a relative weakness of inhibitory supraspinal influences on these neurons as compared to excitatory ones. In the overwhelming majority of neurons, cooling did not affect their phase of response, suggesting that these neurons belong to the networks generating the spinal component of PLRs, and that supraspinal postural commands strongly affect these neurons. In 19% of neurons non-modulated before cooling, the modulation appeared during cooling, suggesting that supraspinal influences reduce activity in the reflex arcs transmitting somatosensory information to these neurons, and thus affected processing of sensory information in the spinal cord. The proportion of F-neurons inactivated during cooling was significantly larger than found in E-neurons (79% vs. 48%), suggesting that excitatory supraspinal drive to F-neurons is considerably stronger than to E-neurons, which can explain an increase in extensor activity and enhancement of PLRs. In the activated and inactivated F- and E-neurons, cooling affected both the mean burst frequency and mean interburst frequencies (**Figure 7F**), suggesting that most neurons received, respectively, inhibitory and excitatory supraspinal drive during both phases of the tilt cycle. A population of F-neurons residing in the ventromedial part of the gray matter was revealed, which exhibited a dramatic (>80%) decrease in their activity during cooling. It was suggested that elimination of the excitatory supraspinal drive to these neurons is responsible for disappearance of extensor tone and PLRs during spinal shock (Zelenin et al., 2013).

To reveal the spinal pathways critically important for maintenance of lateral stability, lesion studies were performed in rabbits (Lyalka et al., 2005, 2009, 2011). After lateral or dorsal hemisection of the spinal cord at T12, postural corrective responses to lateral tilts recovered in 1–3 weeks, whereas after the ventral hemisection they disappeared completely and did not recover. These findings suggest that RS and vestibulospinal pathways descending in the ventral quadrants are crucially important for the generation of postural reactions.

### TONIC SUPRASPINAL DRIVE

One of the important functions of supraspinal systems is to provide tonic drive to spinal postural networks necessary for their activation. One of the sources of tonic activity of different descending systems (vestibulospinal, RS, etc.) is unspecific tonic inflow from the continuously firing vestibular afferents, which affects them through the vestibular nuclei. Activated by this tonic inflow, the vestibulospinal drive determines a high level of excitability of EMNs during standing and, therefore, a high tonus in the extensor muscles, which is a necessary condition for supporting the body during standing (Duysens et al., 2000), as well as for generation of postural corrections.

Recently, the effects of manipulation with tonic supraspinal drive by means of galvanic vestibular stimulation (GVS) on the postural system were studied (Hsu et al., 2012). The GVS excites and inhibits vestibular afferents on the side of the negative (cathode) and positive (anode) electrode, respectively, (Goldberg et al., 1984; Minor and Goldberg, 1991). Thus the left/right asymmetry in tonic supraspinal drive is created, which results in a lateral body sway toward the anode observed in all studied species including humans (e.g., Séverac Cauquil et al., 2000; Beloozerova et al., 2003a; Gorgiladze, 2004). Analysis of GVS effects in humans shows that the sway is caused mainly by activation of canal afferents (Mian et al., 2010), its direction and amplitude depend on the polarity and strength of the current stimulating the left and right labyrinths, as well as on the initial subject’s posture (Marsden et al., 2002). A model of GVS effects was proposed (Day et al., 2011).

**FIGURE 8:**
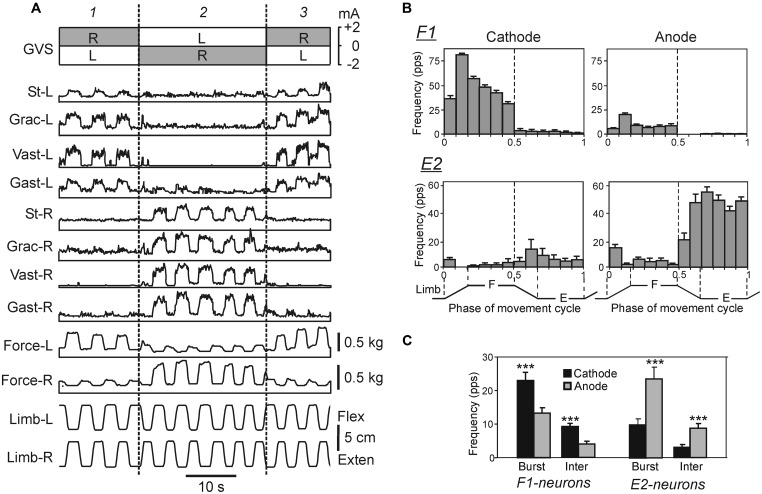
**Effects of galvanic vestibular stimulation on postural limb reflexes and on spinal neurons presumably mediating these reflexes. (A)** An example of GVS effects on PLRs. The configuration of GVS was repetitively changed, so that the anode was on the right side (R) and cathode was on the left side (L) during time periods *1*, and *3*; the position of anode and cathode was opposite during period *2*. The reflex responses of the limb (the EMG value in eight tested muscles and the force magnitude) were much larger when the cathode was ipsilateral to the limb than when the anode was ipsilateral to this limb. St, semitendinosus; Grac, gracilis; Gast, gastrocnemius. **(B)** Examples of the neurons from F1 and E2 subgroups presumably mediating the effects of GVS on PLRs. For each neuron, a histogram of its activity in the F/E cycle of the ipsilateral limb was obtained under two conditions, with ipsilateral cathode and with ipsilateral anode. In F1 neurons, the activity was significantly higher with ipsilateral cathode than with ipsilateral anode. In E2 neurons, the activity was significantly higher with ipsilateral anode than with ipsilateral cathode. **(C)** The effects of GVS on the mean burst and mean interburst frequencies of F1 and E2 neurons under two conditions: with ipsilateral anode (gray bars) and with ipsilateral cathode (black bars). ^∗∗∗^*p* < 0.001, *t*-test.

In the standing rabbit, the GVS-caused new body orientation is actively stabilized (Beloozerova et al., 2003a) due to the change in the set-point of the postural system. The GVS strongly affects the magnitude of PLRs (**Figure 8A**): the extensor EMGs and the force developed during limb flexion are considerably increased when the cathode is ipsilateral to the limb, and decreased when the anode is ipsilateral to the limb (Hsu et al., 2012). Thus, GVS, by creating asymmetry in the tonic left and right supraspinal drive, changes the set-point of the postural system through the change of the gain in antagonistic PLRs. It was also demonstrated that in the caudally decerebrated rabbit, an artificial feedback based on GVS could restore normal postural reactions and lateral stability (Zelenin et al., 2012).

Similar results were obtained in humans: an artificial GVS-based feedback considerably improved lateral stability during standing (Scinicariello et al., 2001). These results suggest similarity in organization of the system responsible for balance during standing in humans and quadrupeds.

The effects of GVS on the activity of spinal interneurons mediating PLRs were analyzed (Hsu et al., 2012). It was shown that asymmetry in the tonic supraspinal drive (caused by GVS) produces diverse effects on the activity of individual F- and E-neurons. Two sub-groups of spinal interneurons presumably mediating the effect of GVS on PLRs were found. The activity in F1-neurons increased with cathodal GVS and decreased with anodal GVS (**Figures 8B,C**), as the activity of EMNs. By contrast, E2-neurons exhibited responses to GVS that were opposite to those in EMNs (**Figures 8B,C**). It was suggested that the F1 and E2 neurons regulate the degree of activation and inactivation of EMNs during PLRs, respectively, in accordance with supraspinal drive (determined by the GVS polarity). Neurons of F1 and E2 subgroups are located mainly in the intermediate and ventral part of the gray matter, respectively, that is in the areas of termination of the vestibulospinal tract (Nyberg-Hansen and Mascitti, 1964; Petras, 1967), and thus can receive direct vestibulospinal influences.

**FIGURE 9:**
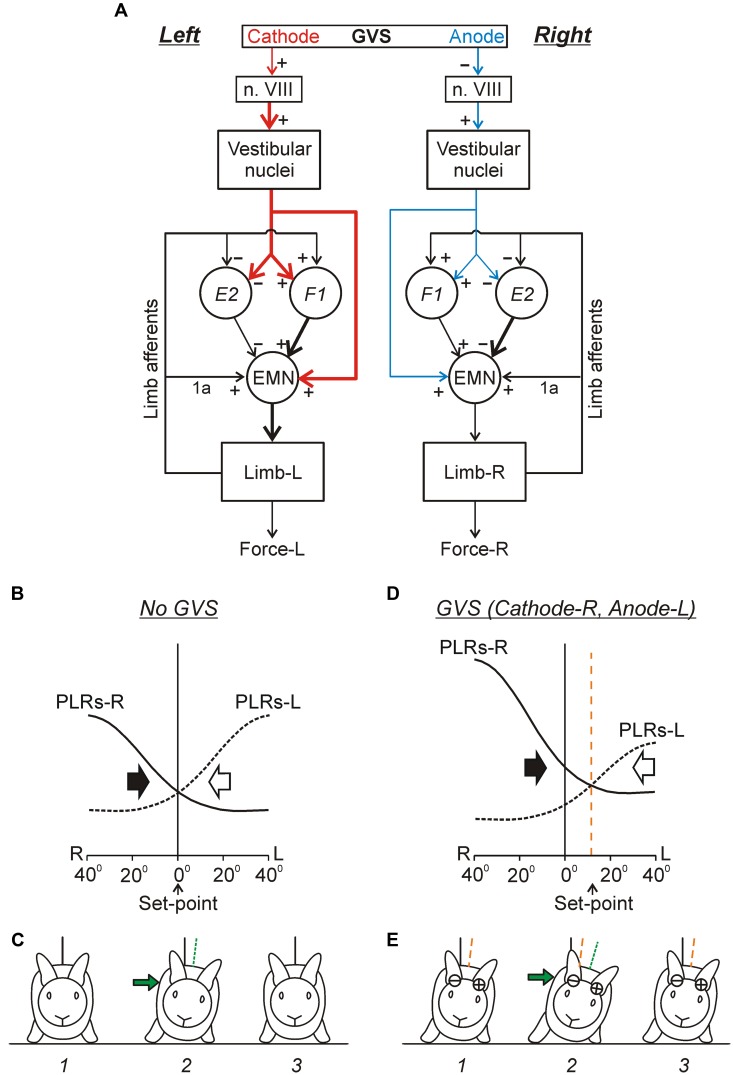
**Conceptual model of the trunk stabilization system and effects of galvanic vestibular stimulation. (A)** Schematic representation of two chains of PLRs (Left and Right), as well as the effects of GVS on these chains. In each chain, flexion/loading of the limb activates afferents of this limb. They excite extensor motoneurons (EMNs) through monosynaptic pathways (group 1a afferents) and through polysynaptic pathways mediated by spinal interneurons (groups F1 and E2). Extensor motoneurons activate extensor muscles, which counteract the limb flexion. The GVS causes asymmetry of the two chains (indicated by different size and thickness of the corresponding red and blue arrows). With cathode on the left side, GVS activates vestibular afferents in the left VIII nerve (n. VIII), which activate neurons of the left vestibular nuclei. These neurons, through the left vestibulospinal tract, affect the spinal postural reflexes on the left side (for simplicity, crossed-effects are not considered). Due to this changed descending drive, excitability of extensor motoneurons and F1-interneurons is increased, and excitability of E2-interneurons is decreased (as compared to the right side). **(B–E)** Presumed effects of the two antagonistic reflex chains in the unrestrained standing rabbit, without GVS **(B,C)** and during GVS with cathode-R and anode-L **(D,E)**. **(B,D)** The abscissa shows a deviation of the dorso-ventral body axis from the vertical (lateral sway); the ordinate shows the value of PLR-R and PLR-L (solid and interrupted line, respectively). Black and white arrows indicate the motor effect (lateral sway) caused by PLR-R and PLR-L, respectively. **(C,E)** The stabilized orientation (1), a deviation due to a lateral push (2), and the restored orientation (3). The stabilized body orientation and the body orientation immediately after the push are indicated by the orange and green interrupted lines, correspondingly. (See text for details).

Two chains of antagonistic PLRs, as well as the effects of GVS on these chains are schematically shown in **Figure 9A**. This scheme reflects also an important finding (Grillner and Hongo, 1972) that the vestibulospinal tract can excite the EMNs both directly and indirectly, through spinal interneurons (presumably subgroups F1 and E2) that integrate descending and afferent information. **Figures 9B–E** illustrates presumed effects of the two antagonistic reflex chains in the unrestrained standing rabbit. The effects without GVS are shown in **Figure 9B**. Any deviation of the dorso-ventral body axis from the vertical (lateral sway) causes opposite changes in PLR-R and PLR-L (solid and interrupted lines, respectively). In turn, PLR-R and PLR-L produce opposite motor effects – they cause body sway in opposite directions as indicated by black and white arrows, respectively. With symmetrical PLRs (as in **Figure 9B**), the two curves intersect at 0° (no lateral sway). This orientation (**Figure 9C**, 1) is stabilized, i.e., the rabbit will return to this orientation after any deflection caused, e.g., by the lateral push (**Figure 9C**, 2 and 3).

Continuous GVS (e.g., with Cathode-R, Anode-L) causes an increase in PLR-R and a decrease in PLR-L (**Figure 9D**). Now the two curves intersect not at 0° but at some angle of the left sway. This tilted orientation (**Figure 9E**, 1) will be stabilized, i.e., the rabbit will return to this orientation after any deflection from it (caused, e.g., by lateral push, **Figure 9E**, 2 and 3). Thus, GVS changes the set-point in the control system. A similar principle of balance control, as well as a similar mechanism underlying a change of stabilized orientation were found in simpler animals – a mollusk (*Clione*) and a lower vertebrate, lamprey (**Figures 5A–C**; Deliagina et al., 1998, 2006b; Deliagina and Fagerstedt, 2000).

As in the lamprey, the immediate effect of UL in higher vertebrates is the loss of lateral stability, and continuous rolling toward the damaged side (e.g., Smith and Curthoys, 1989; Deliagina et al., 1997). As in the lamprey, electrical stimulation of the vestibular nerve terminates rolling and restores lateral stability in the rat. By changing the strength of stimulation, the stabilized body orientation in the transverse plane can be regulated (Deliagina et al., 1997). One can suggest that as in the lamprey (**Figure 5D**) UL causes strong asymmetry in the tonic supraspinal drive. This leads to a dramatic decrease in the gain of PLRs on the damaged side, resulting in disappearance of a set-point in the postural system operating in the transverse plane. The activity of PLR network on the intact side leads to rolling toward the damaged side. Electrical stimulation of the vestibular nerve restores the symmetry in supraspinal drive. This results in an increase in the gain of PLRs on the damaged side, re-appearance of the set-point of the system, and restoration of the lateral stability.

One can expect that in humans the principles of operation of the postural system responsible for stabilization of the body orientation in the frontal plane is similar to that revealed in animal models, and a lateral body sway caused by GVS (Séverac Cauquil et al., 2000) can be explained by a shift of the equilibrium point of the control system.

### PHASIC SUPRASPINAL POSTURAL COMMANDS

#### Reticulospinal system

The activity of RS neurons during postural reactions to drop of support under one of the limbs was analyzed in the cat (Stapley and Drew, 2009). In the standing cat, this perturbation produces postural reactions, which result in transition from quadrupedal to tripedal standing (Dufossé et al., 1985; Rushmer et al., 1987; Stapley and Drew, 2009). The initial postural changes in the supporting limbs are caused by sensory information from the dropping limb (Stapley and Drew, 2009). The majority of RS neurons respond to this perturbation with a short latency preceding the initial change in EMGs, suggesting that their discharge represents a postural command contributing to initiation of the postural corrective reaction.

The striking result is that only about 10% of neurons respond to drop of only one of the limbs, suggesting that they encode a command contributing to initiation of only one specific postural reaction. The majority of RS neurons respond to drop of different limbs, thus contributing to generation of different specific postural reactions.

About three quarters of the RS neurons are activated by perturbation of any of two or three limbs. Drop of the support under one of the limbs causes a specific disturbance of body orientation in both pitch and roll planes. One may hypothesize that, as in the lamprey, individual RS neurons in the cat produce motor output contributing to generation of postural correction in a particular vertical plane. For example, RS neurons contributing to rotation of the trunk to the left in the roll plane will be activated by drop of the surface under right forelimb and right hindlimb, and inhibited by drop of the surface under left forelimb and left hindlimb. Neurons with reciprocal responses to the right and left perturbations of the trunk orientation comprised about 25% of the RS population.

Finally, about 15% of RS neurons are activated by the support drop under any of the limbs. One can suggest that these RS neurons generate a “GO” command, and the motor response to this command depends on the current state of spinal networks affected by specific supraspinal and somatosensory inputs.

Thus, the study by Stapley and Drew (2009) has clearly demonstrated that RS neurons may contribute to the compensatory postural reactions that follow an unexpected perturbation. This study also presented arguments against a contribution of the RS system to the specification of the detailed postural reaction required for the compensation. This role most likely belongs to the corticospinal and rubrospinal systems.

#### Corticospinal and rubrospinal systems

**FIGURE 10:**
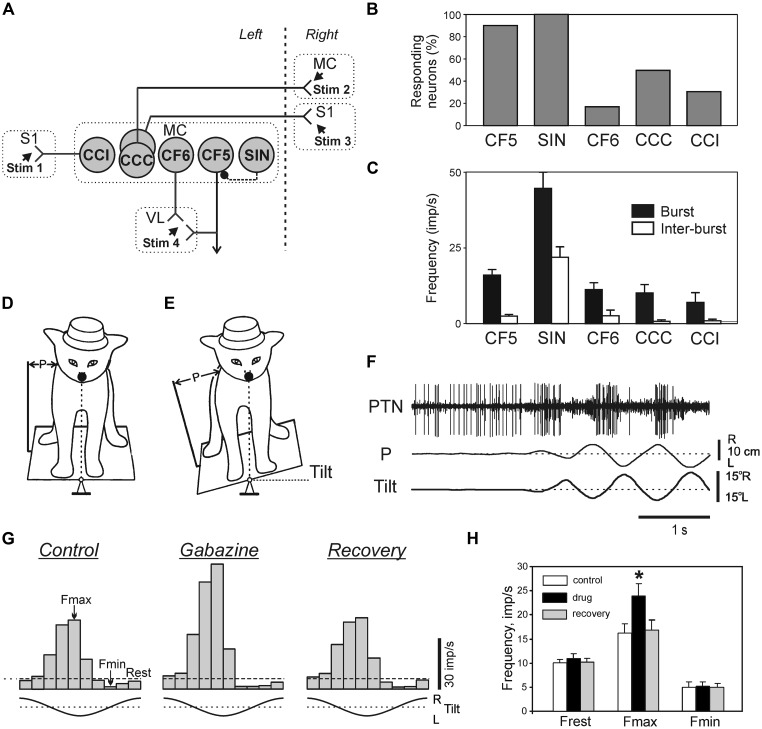
**Activity of different classes of neurons in the motor cortex during postural corrections caused by lateral tilts of the platform. (A)** Types of neurons which were recorded in the forelimb representation of the left motor cortex (MC) in rabbits. CCI, cortico-cortical neurons projecting to the ipsilateral primary somatosensory cortex (S1); CCC, cortico-cortical neurons projecting to the contralateral motor (MC) or primary somatosensory cortex (S1); CF6, corticofugal neurons of layer VI projecting to the ventrolateral thalamus (VL); CF5, corticofugal neurons of layer V with collaterals projecting to the ventrolateral thalamus (these neuron types were identified by their antidromic responses to electrical stimulation of the corresponding structures, Stim 1–Stim 4); SIN, putative inhibitory interneurons (identified by their high-frequency orthodromic responses to stimulation of ventralateral thalamus or a cortical site). **(B)** Proportion of neurons responding to tilts in different classes of cortical neurons. **(C)** Mean burst and mean interbust frequencies of modulated neurons in different classes of cortical neurons. **(D,E)** Experimental design for recording the activity of pyramidal tract neurons (PTNs) during postural corrections. PTN activity was recorded along with platform tilts (Tilt) and postural corrections (P). **(F)** An example of PTN responses to tilts. **(G)** The effect of gabazine on postural responses of a PTN. Histograms of the PTN activity during tilts for each of three conditions: Control, before application; Gabazine, 2 min after gabazine application; Recovery, 15 min after application. Fmax and Fmin, the maximum and minimum frequencies in the histogram. **(H)** Effects of GABA-A receptor antagonists on PTN population activity: Frest, Fmax, and Fmin. ^∗^*p* < 0.05, *t*-test.

Despite the fact that integrity of the cerebral cortex is not critical for the ability to maintain lateral stability (Musienko et al., 2008), in intact animals and humans the cortical mechanisms supplement the basic brainstem-cerebellum-spinal cord mechanisms during maintenance of the basic body posture (for review see, e.g., Jacobs and Horak, 2007). Recording activity of different classes of neurons (**Figure 10A**) of the motor cortex (MC) in awake rabbits, while the animals maintained balance on a laterally tiling platform, have shown that activity of descending corticofugal neurons of layer V (CF5s) [which includes pyramidal tract neurons (PTNs)] and one class of GABA-ergic inhibitory interneurons (SINs) was strongly correlated to the postural corrections (Beloozerova et al., 2003b; **Figures 10B,C**). In contrast to CF5 and SINs, the proportion of corticofugal neurons of layer VI (CF6s) and of cortico-cortical neurons with ipsilateral (CCIs) and cortico-cortical neurons with contralateral (CCCs) projections that were active during postural corrections was relatively small (**Figure 10B**), and their discharge frequencies were low (**Figure 10C**). This suggests that cortico-cortical interactions, both within a hemisphere (mediated by CCIs) and between hemispheres (mediated by CCCs), as well as cortico-thalamic interactions via CF6 neurons are not essential for motor coordination during postural corrections.

The tilt-related signals from the spinal cord and brainstem can reach the MC and affect its output neurons (CF5) via different routes. One of these is an input via ventro-lateral thalamus, a part of which is mediated by SINs (Strick and Sterling, 1974; White, 1989; Swadlow, 2002). Since activity of SINs is rhythmically modulated during postural corrections, one can hypothesize that they contribute to shaping of cortical output. The activity of individual PTNs in the cat maintaining balance on a tilting platform (**Figures 10D,F**) was recorded both before and after local iontophoretic application of the GABA-A receptor antagonists at the site of recording (Tamarova et al., 2007). It was found that the GABA-ergic system of the MC attenuates the posture-related responses of PTNs but plays little role in determining the response timing (**Figures 10G,H**).

Activity of individual neurons of two supraspinal systems (corticospinal and rubrospinal) was studied in awake cats maintaining balance on the tilting platform (**Figures 10D,E**; Beloozerova et al., 2005; Karayannidou et al., 2008, 2009b; Zelenin et al., 2010). It was found that activity of these two systems in the postural task has many features in common.

*First,* a considerable proportion of neurons in both systems are phasically modulated by tilts (**Figure 10F**), though the proportion of modulated rubral neurons is smaller (46%) than the cortical ones (90%). Modulated PTNs and rubrospinal neurons (RbNs) can be forelimb- or hindlimb-related. A half of PTNs have a positional response to tilt, i.e., their activity depends on the value of stationary tilt. Taken together these results suggest that the MC and red nucleus send postural commands to the spinal cord and medulla. The MC along with other descending systems including reticulo- and vestibulospinal ones (Matsuyama and Drew, 2000), participates in execution of both principal postural functions: the maintenance of a definite body configuration and the maintenance of equilibrium (Horak and Macpherson, 1996).

*Second*, in both corticospinal and rubrospinal systems, the phases of activity of individual neurons were distributed over the entire tilt cycle, and the role of RbNs and PTNs in the postural task is difficult to assess on the basis of a simple correlation between the population activity and the motor pattern.

**FIGURE 11:**
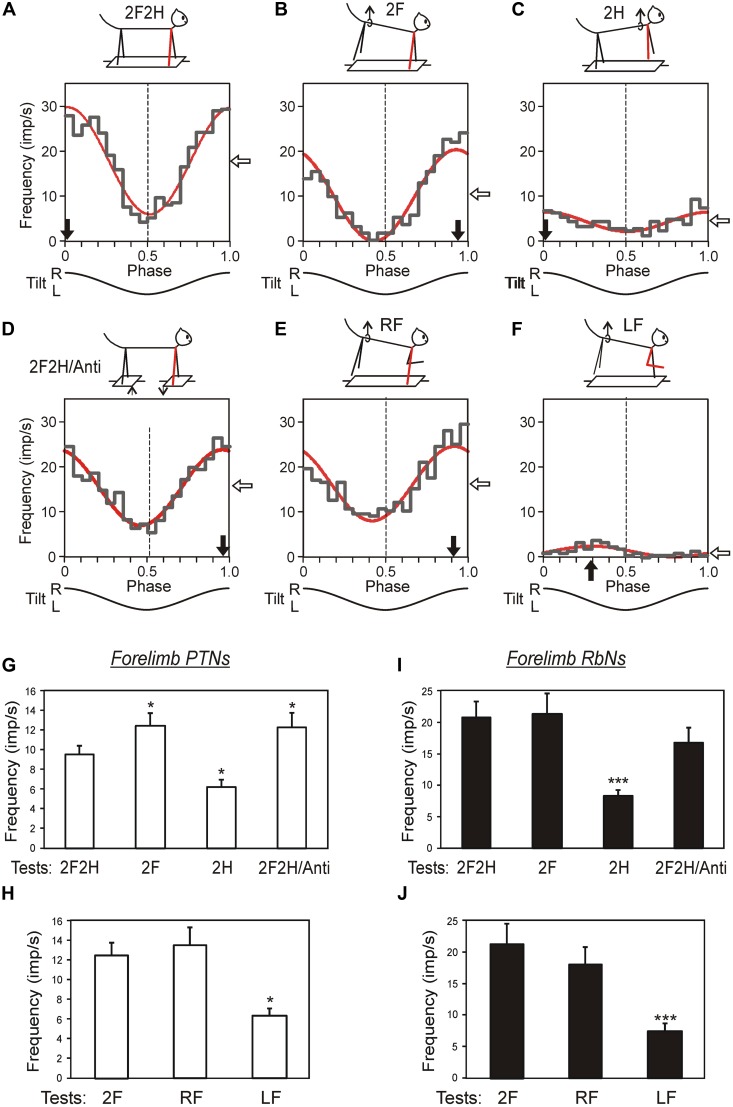
**Activity of corticospinal and rubrospinal neurons in the cat during postural corrections. (A–F)** Activity of forelimb-related RbS neuron from left red nucleus during different postural tests. The neuron was related to the right forelimb (indicated by blue). **(A)** Control, standing on all four limbs (test 2F2H). **(B)** Standing on two forelimbs (test 2F). **(C)** Standing on two hindlimbs (test 2H). **(D)** Antiphase tilts of the platforms under the forelimbs and hindlimbs (test 2F2H/Anti). **(E)** Standing on the right forelimb (test RF). **(F)** Standing on the left forelimb (test LF). For each test the following are shown: (1) the phase histogram of spike activity in the tilt cycle (gray line), (2) the first harmonic of Fourier image (red line), (3) the mean frequency of discharge (white arrow), and (4) the preferred phase (black arrow). **(G–J)** Population characteristics of forelimb PTNs **(G,H)** and RbSNs **(I,J)** in tests revealing influences from shoulder and hip girdles **(G,I)**, and in tests revealing influences from individual limbs of the same girdle **(H,J)**. Mean value of modulation depth, that is the peak-to-peak value of the first harmonic, is shown. ^∗^*p* < 0.05, ^∗∗∗^*p* < 0.001, *t*-test.

*Third,* the contribution of tilt-related sensory inputs from individual limbs to posture-related modulation of individual RbNs and PTNs was examined by eliminating tilt-related sensory input from one, two or three limbs (**Figures 11A–F**). In the presented example, the forelimb-related RbN from the left red nucleus has the same phase and depth of modulation in all those tests in which the right forelimb is standing on the tilting platform, and thus tilt-related somatosensory input from this limb is present. The amplitude and phase of responses to platform tilts in the majority of RbNs and PTNs are determined primarily by sensory input from the corresponding (fore or hind) contralateral limb, whereas inputs from the other limbs make a much smaller contribution to their modulation (**Figures 11G–J**). Thus, in the sub-systems responsible for stabilization of the anterior and posterior parts of the trunk in the transverse plane, PTNs and RbNs are elements of the feedback mechanism generating corrective limb movement on the basis of sensory information from the same limb (**Figure 6D**).

*Fourth*, in the majority of PTNs and RbNs, the afferent signals that they presumably receive from their receptive fields during tilts cannot be even partially responsible for the generation of neuronal reactions to tilts (Beloozerova et al., 2003b, 2005; Zelenin et al., 2010). Most likely, in these neurons the somatosensory input from the receptive field determined at rest, is replaced or complemented by other inputs during active postural behavior. This hypothesis is further supported by the view that the signals from limb mechanoreceptors are processed in the spinal and brainstem networks before they reach the MC, and in the cerebellum and MC before they reach rubral neurons (Massion, 1967; Toyama et al., 1968; Landgren and Silfvenius, 1971; Asanuma, 1989).

Thus, in quadrupeds, all studied descending tracts transmit postural commands to the spinal cord. One can expect that postural commands in humans are also transmitted through many descending pathways.

## CONCLUDING REMARKS

During the last decade, the use of different animal models and novel techniques has enabled considerable progress to be made in understanding the functional organization of postural mechanisms and in analysis of the underlying neuronal networks. Some differences in organization but remarkable similarities in principles of operation of the postural control system in the lamprey and in the quadrupeds have been revealed.

(1) The postural system in the lamprey and quadrupeds responds to numerous destabilizing factors by producing specific postural corrections. These corrections in the lamprey and quadrupeds are caused by different types of sensory information – vestibular in the lamprey and mainly somatosensory (from limb mechanoreceptors) in quadrupeds. This difference reflects different environmental conditions for aquatic and terrestrial animals.
(2) In the lamprey, there is only one system that controls the postural orientation of the whole body. In quadrupeds, the trunk stabilization system can dissociate into two independent sub-systems controlling orientation of the anterior and posterior body parts. This is important in the cases of complex configuration of the support surface, but irrelevant for the lamprey living in a homogeneous medium (water).
(3) In both lampreys and quadrupeds, stabilization of body orientation in the transverse plane is based on the interaction of two antagonistic reflexes (vestibulospinal reflexes in the lamprey and PLRs in quadrupeds). The animal stabilizes its orientation at the point at which these reflexes are equal to each other.
(4) In both lampreys and quadrupeds, these antagonistic reflexes are mediated by neurons of supraspinal systems. Phasic postural commands, transmitted by supraspinal neurons to the spinal cord, play a crucial role in the generation of postural corrections. In the lamprey, supraspinal commands are responsible for elicitation of postural corrections, and the role of spinal networks is transformation of these commands into an appropriate motor pattern. In quadrupeds, this mechanism also exists but it is supplemented with spinal postural networks (generating spinal PLRs), which are regulated by the supraspinal tonic drive. One of the lines of future studies is the analysis of operation of spinal neuronal networks in different postural tasks, as well as search for the factors enhancing their efficacy in subjects with spinal cord injury. Another line is to understand how the capability of the spinal cord for sophisticated processing of somatosensory information is used in postural mechanisms.
(5) In both lampreys and quadrupeds, the stabilized body orientation can be changed through a change of the gain in antagonistic reflex chains, which causes a shift of the equilibrium point of the control system. In both lamprey and quadrupeds, supraspinal mechanisms are responsible for this function. In the lamprey, the neuronal mechanisms underlying the shift of the equilibrium point were revealed. The goal of future studies is to reveal these mechanisms in quadrupeds.
(6) In the lamprey, postural commands to the spinal cord are transmitted by the only developed descending system, the RS one. The mechanisms of encoding and decoding of these commands have been revealed. In quadrupeds, postural commands are transmitted by many descending pathways. In a few examined postural tasks, phasic postural commands transmitted by corticospinal, rubrospinal, and RS systems were analyzed, and a difference in function has been revealed between corticospinal and RbNs on one hand, and RS neurons on the other. The goal of future studies is to understand the neuronal mechanisms of formation of supraspinal postural commands in quadrupeds, as well as their processing by the spinal networks, which results in the corrective motor response.

## Conflict of Interest Statement

The authors declare that the research was conducted in the absence of any commercial or financial relationships that could be construed as a potential conflict of interest.

## References

[B1] Ackermann H. Dichgans J. Guschlbauer B. (1991). Influences of an acoustic preparatory signal on postural reflexes of the distal leg muscles in humans. *Neurosci. Lett.* 127 242–246 10.1016/0304-3940(91)90803-2 1881636

[B2] Ackermann H. Dichgans J. Guschlbauer B. Scholz E. (1990). “Postural modulation of evoked cortical and motor potentials and its relationship to functional adaptation of postural reflexes,” in *Disorders of Posture and Gait* eds Brandt T. Paulus W. Bles W. Dieterich M. Krafczyk S. Straube A. (Stuttgart: Thieme) 86–89

[B3] Archambault P. S. Deliagina T. G. Orlovsky G. N. (2001). Non-undulatory locomotion in the lamprey. *Neuroreport* 12 1803–1807 10.1097/00001756-200107030-00009 11435902

[B4] Asanuma H. (1989). *The Motor Cortex.* New York: Raven Press

[B5] Beloozerova I. N. Sirota M. G. Orlovsky G. N. Deliagina T. G. (2005). Activity of pyramidal tract neurons in the cat during postural corrections. *J. Neurophysiol.* 93 1831–1844 10.1152/jn.00577.2004 15525811

[B6] Beloozerova I. N. Zelenin P. V. Popova L. B. Orlovsky G. N. Grillner S. Deliagina T. G. (2003a). Postural control in the rabbit maintaining balance on the tilting platform. *J. Neurophysiol.* 90 3783–3793 10.1152/jn.00590.2003 12930819

[B7] Beloozerova I. N. Sirota M. G. Swadlow H. A. Orlovsky G. N. Popova L. B. Deliagina T. G. (2003b). Activity of different classes of neurons of the motor cortex during postural corrections. *J. Neurosci.* 23 7844–7853 12944514 10.1523/JNEUROSCI.23-21-07844.2003 PMC6740594

[B8] Berger W. Horstmann G. A. Dietz V. (1990). Interlimb coordination of stance in children: divergent modulation of spinal reflex responses and cerebral evoked potantials in terms of age. *Neurosci. Lett.* 116 118–122 10.1016/0304-3940(90)90396-Q 2259442

[B9] Bouisset S. Do M.-C. (2008). Posture, dynamic stability, and voluntary movement. *Clin. Neurophysiol.* 38 345–362 10.1016/j.neucli.2008.10.001 19026956

[B10] Bussières N. (1994). *Les Systemes Descendants chez la Lamproie. Etude Anatomique et Functionnelle.* Montreal: University of Montreal

[B11] Carpenter M. G. Allum J. H. J. Honegger F. (1999). Directional sensitivity of stretch reflexes and balance corrections for normal subjects in the roll and pitch planes. *Exp. Brain Res.* 129 93–113 10.1007/s002210050940 10550507

[B12] Carpenter M. G. Allum J. H. J. Honegger F. (2001). Vestibular influences on human postural control in combinations of pitch and roll planes reveal differences in spatiotemporal processing. *Exp. Brain Res.* 140 95–111 10.1007/s002210100802 11500802

[B13] Chvatal S. A. Macpherson J. M. Torres-Oviedo G. Ting L. H. (2013). Absence of postural muscle synergies for balance after spinal cord transection. *J. Neurophysiol.* 110 1301–1310 10.1152/jn.00038.2013 23803327 PMC3763149

[B14] Cullen K. E. (2012). The vestibular system: multimodal integration and encoding of self-motion for motor control. *Trends Neurosci.* 35 185–196 10.1016/j.tins.2011.12.001 22245372 PMC4000483

[B15] Day B. L. Ramsay E. Welgampola M. S. Fitzpatrick R. C. (2011). The human semicircular canal model of galvanic vestibular stimulation. *Exp. Brain Res.* 210 561–568 10.1007/s00221-011-2565-7 21287152 PMC3075401

[B16] Deliagina T. G. (1995). Vestibular compensation in the lamprey. *Neuroreport* 6 2599–2603 10.1097/00001756-199512150-00035 8741771

[B17] Deliagina T. G. (1997a). Vestibular compensation in lampreys: impairment and recovery of equilibrium control during locomotion. *J. Exp. Biol.* 200 1459–1471 9319361 10.1242/jeb.200.10.1459

[B18] Deliagina T. G. (1997b). Vestibular compensation in lampreys: role of vision at different stages of recovery of equilibrium control. *J. Exp. Biol.* 200 2957–2967 9359882 10.1242/jeb.200.23.2957

[B19] Deliagina T. G. Arshavsky Y. I. Orlovsky G. N. (1998). Control of spatial orientation in a mollusc. *Nature* 393 172–175 10.1038/30251 9603520

[B20] Deliagina T. G. Fagerstedt P. (2000). Responses of reticulospinal neurons in intact lamprey to vestibular and visual inputs. *J. Neurophysiol.* 83 864–878 10669500 10.1152/jn.2000.83.2.864

[B21] Deliagina T. G. Grillner S. Orlovsky G. N. Ullén F. (1993). Visual input affects the response to roll in reticulospinal neurons of the lamprey. *Exp. Brain Res.* 95 421–428 10.1007/BF00227134 8224067

[B22] Deliagina T. G. Orlovsky G. N. (2002). Comparative neurobiology of postural control. *Curr. Opin. Neurobiol.* 12 652–657 10.1016/S0959-4388(02)00376-8 12490255

[B23] Deliagina T. G. Orlovsky G. N. Grillner S. Wallen P. (1992a). Vestibular control of swimming in lamprey. 2. Characteristics of spatial sensitivity of reticulospinal neurons. *Exp. Brain Res.* 90 489–498 10.1007/BF00230931 1426109

[B24] Deliagina T. G. Orlovsky G. N. Grillner S. Wallen P. (1992b). Vestibular control of swimming in lamprey. 3. Activity of vestibular afferents. Convergence of vestibular inputs on reticulospinal neurons. *Exp. Brain Res.* 90 499–507 10.1007/BF00230932 1426110

[B25] Deliagina T. G. Sirota M. G. Zelenin P. V. Orlovsky G. N. Beloozerova I. N. (2006a). Interlimb postural coordination in the standing cat. *J. Physiol.* 573 211–224 10.1113/jphysiol.2006.104893 16527856 PMC1779703

[B26] Deliagina T. G. Orlovsky G. N. Zelenin P. V. Beloozerova I. N. (2006b). Neural bases of postural control. *Physiology (Bethesda)* 21 216–225 10.1152/physiol.00001.2006 16714480

[B27] Deliagina T. G. Pavlova E. L. (2002). Modifications of vestibular responses of individual reticulospinal neurons in the lamprey caused by a unilateral labyrinthectomy. *J. Neurophysiol.* 87 1–14 11784725 10.1152/jn.00315.2001

[B28] Deliagina T. G. Popova L. B. Grant G. (1997). The role of tonic vestibular input for postural control in rats. *Arch. Ital. Biol.* 135 239–261 9177127

[B29] Deliagina T. G. Zelenin P. Fagerstedt P. Grillner S. Orlovsky G. (2000a). Activity of reticulospinal neurons during locomotion in the freely behaving lamprey. *J. Neurophysiol.* 83 853–863 10669499 10.1152/jn.2000.83.2.853

[B30] Deliagina T. G. Beloozerova I. N. Popova L. B. Sirota M. G. Swadlow H. Grant G. (2000b). Role of different sensory inputs for maintenance of body posture in sitting rat and rabbit. *Motor Control* 4 439–452 11020673 10.1123/mcj.4.4.439

[B31] Dietz V. Quintern J. Berger W. (1984). Cerebral evoked potentials associated with the compensatory reactions following stance and gait perturbation. *Neurosci. Lett.* 50 181–186 10.1016/0304-3940(84)90483-X 6493623

[B32] Dietz V. Quintern J. Berger W. Schenck E. (1985). Cerebral potentials and leg muscle e.m.g. responses associated with stance perturbation. *Exp. Brain Res.* 57 354–384 10.1007/BF00236540 3972035

[B33] Dufossé M. Macpherson J. Massion J. (1982). Biomechanical and electromyographical comparison of two postural supporting mechanisms in the cat. *Exp. Brain Res.* 45 38–44 10.1007/BF00235761 7056336

[B34] Dufossé M. Macpherson J. Massion J. Sybirska E. (1985). The postural reaction to the drop of a hindlimb support in the standing cat remains following sensorimotor cortical ablation. *Neurosci. Lett.* 55 297–303 10.1016/0304-3940(85)90452-5 4011034

[B35] Duysens J. Clarac F. Cruse H. (2000). Load-regulating mechanisms in gait and posture: comparative aspects. *Physiol. Rev.* 80 83–133 10617766 10.1152/physrev.2000.80.1.83

[B36] Fung J. Macpherson J. M. (1999). Attributes of quiet stance in the chronic spinal cat. *J. Neurophysiol.* 82 3056–3065 10601441 10.1152/jn.1999.82.6.3056

[B37] Goldberg J. M. Smith C. E. Fernandez C. (1984). Relation between discharge regularity and responses to externally applied galvanic currents in vestibular nerve afferents of the squirrel monkey. *J. Neurophysiol.* 51 1236–1256 6737029 10.1152/jn.1984.51.6.1236

[B38] Gorgiladze G. I. (2004). Electrical stimulation of labyrinths and vestibular reactions. *Bull.* *Exp. Biol. Med*. 138 629–631 10.1007/s10517-005-0143-3 16134830

[B39] Grillner S. Deliagina T. Ekeberg Ö. El Manira A. Hill R. Lansner A. (1995). Neural networks controlling locomotion and body orientation in lamprey. *Trends Neurosci.* 18 270–279 10.1016/0166-2236(95)93914-J 7571002

[B40] Grillner S. Hongo T. (1972). Vestibulospinal effects on motoneurons and interneurons in the lumbosacral cord. *Prog. Brain Res.* 37 244–262 10.1016/S0079-6123(08)63906-0 4642044

[B41] Grillner S. Wallén P. Brodin L. Lansner A. (1991). Neuronal network generating locomotor behavior in lamprey: circuitry, transmitters, membrane properties, and simulation. *Annu. Rev. Neurosci.* 14 169–199 10.1146/annurev.ne.14.030191.001125 1674412

[B42] Henry S. M. Fung J. Horak F. B. (1998). EMG responses to maintain stance during multidirectional surface translations. *J. Neurophysiol.* 80 1939–1950 9772251 10.1152/jn.1998.80.4.1939

[B43] Honeycutt C. F. Cope T. C. Nardelli P. Nichols T. R. (2007). Autogenic spindle pathways mediate the postural response during horizontal support surface perturbations. *Soc. Neurosci. Abstr.* 33 8603.

[B44] Honeycutt C. F. Gottschall J. S. Nichols T. R. (2009). Electromyographic responses from the hindlimb muscles of the decerebrate cat to horizontal support surface perturbations. *J. Neurophysiol.* 101 2751–2761 10.1152/jn.91040.2008 19321638 PMC2694113

[B45] Honeycutt C. F. Nardelli P. Cope T. C. Nichols T. R. (2008). Loss of proprioceptive feedback from muscle disrupts the excitatory postural response to support surface perturbations. *Soc. Neurosci. Abstr.* 34 1817.

[B46] Honeycutt C. F. Nichols T. R. (2010). The decerebrate cat generates the essential features of the force constraint strategy. *J. Neurophysiol.* 103 3266–3273 10.1152/jn.00764.2009 20089811 PMC2888247

[B47] Horak F. Macpherson J. (1996). “Postural orientation and equilibrium,” in *Handbook of Physiology. Exercise: Regulation and Integration of Multiple Systems* eds Shepard J. Rowell L. (New York: Oxford University Press) 255–292

[B48] Hsu L.-J. Zelenin P. V. Grillner S. Orlovsky G. N. Deliagina T. G. (2013). Intraspinal strech receptor neurons mediate different motor respomses along the body in lamprey. *J. Comp. Neurol.* 521 3847–3862 10.1002/cne.23382 23749436

[B49] Hsu L. J. Zelenin P. V. Orlovsky G. N. Deliagina T. G. (2012). Effects of galvanic vestibular stimulation on postural limb reflexes and neurons of spinal postural network. *J. Neurophysiol.* 108 300–313 10.1152/jn.00041.2012 22514291 PMC3434614

[B50] Islam S. S. Zelenin P. V. (2008). Modifications of locomotor pattern underlying escape behavior in the lamprey. *J. Neurophysiol.* 99 297–307 10.1152/jn.00903.2007 18003880

[B51] Islam S. S. Zelenin P. V. Orlovsky G. N. Grillner S. Deliagina T. G. (2006). The pattern of motor coordination underlying backward swimming in the lamprey. *J. Neurophysiol.* 96 451–460 10.1152/jn.01277.2005 16772518

[B52] Jacobs J. V. Horak F. B. (2007). Cortical control of postural responses. *J. Neural Transm.* 114 1339–1348 10.1007/s00702-007-0657-0 17393068 PMC4382099

[B53] Karayannidou A. Zelenin P. V. Orlovsky G. N. Sirota M. G. Beloozerova I. N. Deliagina T. G. (2009a). Maintenance of lateral stability during standing and walking in the cat. *J. Neurophysiol.* 101 8–19 10.1152/jn.90934.2008 19004997 PMC2637002

[B54] Karayannidou A. Beloozerova I. N. Zelenin P. V. Stout E. E. Sirota M. G. Orlovsky G. N. (2009b). Activity of pyramidal tract neurons in the cat during standing and walking on an inclined plane. *J. Physiol.* 587 3795–3811 10.1113/jphysiol.2009.170183 19491244 PMC2746611

[B55] Karayannidou A. Deliagina T. G. Tamarova Z. A. Sirota M. G. Zelenin P. V. Orlovsky G. N. (2008). Influences of sensory input from the limbs on feline corticospinal neurons during postural responses. *J. Physiol.* 586 247–263 10.1113/jphysiol.2007.144840 17974591 PMC2375550

[B56] Karayannidou A. Zelenin P. V. Orlovsky G. N. Deliagina T. G. (2007). Responses of reticulospinal neurons in the lamprey to lateral turns. *J. Neurophysiol.* 97 512–521 10.1152/jn.00912.2006 17079339

[B57] Landgren S. Silfvenius H. (1971). Nucleus Z, the medullary relay in the projection path to the cerebral cortex of group I muscle afferents from the cat’s hind limb. *J. Physiol.* 218 551–571 4109115 10.1113/jphysiol.1971.sp009633 PMC1331601

[B58] Lowenstein O. Osborne M. R. Thornhill R. A. (1968). The anatomy and ultrastructure of the labyrinth of the lamprey (*Lamperta fluviatilis* L.). *Proc. R. Soc. Lond. B Biol. Sci.* 170 113–134 10.1098/rspb.1968.0029 4385240

[B59] Lyalka V. F. Hsu L.-J. Karayannidou A. Zelenin P. V. Orlovsky G. N. Deliagina T. G. (2011). Facilitation of postural limb reflexes in spinal rabbits by serotonergic agonist administration, epidural electrocal stimulation, and postural training. *J. Neurophysiol.* 106 1341–1354 10.1152/jn.00115.2011 21653706

[B60] Lyalka V. F. Orlovsky G. N. Deliagina T. G. (2009). Impairment of postural control in rabbits with extensive spinal cord lesions. *J. Neurophysiol.* 101 1932–1940 10.1152/jn.00009.2008 19164112 PMC2695648

[B61] Lyalka V. F. Zelenin P. V. Karayannidou A. Orlovsky G. N. Grillner S. Deliagina T. G. (2005). Impairment and recovery of postural control in rabbits with spinal cord lesions. *J. Neurophysiol.* 94 3677–3690 10.1152/jn.00538.2005 16049143

[B62] Macpherson J. M. (1988a). Strategies that simplify the control of quadrupedal stance. I. Forces at the ground. *J. Neurophysiol.* 60 204–217 3404217 10.1152/jn.1988.60.1.204

[B63] Macpherson J. M. (1988b). Strategies that simplify the control of quadrupedal stance. II. Electromyographic activity. *J. Neurophysiol.* 60 218–231 3404218 10.1152/jn.1988.60.1.218

[B64] Macpherson J. M. Everaert D. G. Stapley P. J. Ting L. H. (2007). Bilateral vestibular loss in cats leads to active destabilization of balance during pitch and roll rotations of the support surface. *J. Neurophysiol.* 97 4357–4367 10.1152/jn.01338.2006 17428912

[B65] Macpherson J. M. Fung J. (1999). Weight support and balance during perturbed stance in the chronic spinal cat. *J. Neurophysiol.* 82 3066–3081 10601442 10.1152/jn.1999.82.6.3066

[B66] Marsden J. F. Castellote J. Day B. L. (2002). Bipedal distribution of human vestubular-evoked postural responses during asymmetrical standing. *J. Physiol.* 542 323–331 10.1113/jphysiol.2002.019513 12096073 PMC2290383

[B67] Massion J. (1967). The mammalian red nucleus. *Physiol. Rev.* 47 383–436 4864540 10.1152/physrev.1967.47.3.383

[B68] Massion J. (1998). Postural control systems in developmental perspective. *Neurosci. Biobehav. Rev.* 22 465–472 10.1016/S0149-7634(97)00031-6 9595556

[B69] Massion J. Alexandrov A. Frolov A. (2001). Why and how are posture and movement coordinated? *Prog. Brain Res.* 143 13–27 10.1016/S0079-6123(03)43002-1 14653147

[B70] Matsuyama K. Drew T. (2000). Vestibulospinal and reticulospinal neuronal activity during locomotion in the intact cat. II. Walking on an inclined plane. *J. Neurophysiol.* 84 2257–2276 11067970 10.1152/jn.2000.84.5.2257

[B71] Mian O. S. Dakin C. J. Blouin J. S. Fitzpatrick R. C. Day B. L. (2010). Lack of otolith involvement in balance responses evoked by mastoid electrocal stimulation. *J. Physiol.* 588 4441–4451 10.1113/jphysiol.2010.195222 20855437 PMC3008850

[B72] Minor L. B. Goldberg J. M. (1991). Vestibular-nerve inputs to the vestibulo-ocular reflex: a functional-ablation study in the squirrel monkey. *J. Neurosci.* 11 1636–1648 2045879 10.1523/JNEUROSCI.11-06-01636.1991 PMC6575423

[B73] Mori S. (1987). Integration of posture and locomotion in acute decerebrate cats and in awake, freely moving cats. *Prog. Neurobiol.* 28 161–196 10.1016/0301-0082(87)90010-4 3544055

[B74] Musienko P. E. Deliagina T. G. Gerasimenko Y. P. Orlovsky G. N. Zelenin P. V. (2014). Limb and trunk mechanisms for balance control during locomotion. *J. Neurosci.* 34 5704–5716 10.1523/JNEUROSCI.4663-13.2014 24741060 PMC3988419

[B75] Musienko P. E. Zelenin P. V. Lyalka V. F. Orlovsky G. N. Deliagina T. G. (2008). Postural performance in decerebrated rabbit. *Behav. Brain Res.* 190 124–134 10.1016/j.bbr.2008.02.011 18359100 PMC2365477

[B76] Musienko P. E. Zelenin P. V. Orlovsky G. N. Deliagina T. G. (2010). Facilitation of postural limb reflexes with epidural stimulation in spinal rabbits. *J. Neurophysiol.* 103 1080–1092 10.1152/jn.00575.2009 20018835 PMC2822695

[B77] Nieuwenhuys R. Ten Donkelaar H. R. (1996). *The Central Nervous System of Vertebrates.* Berlin: Springer

[B78] Nyberg-Hansen R. Mascitti T. A. (1964). Sites and mode of termination of fibers of the vestibulospinal tract in the cat. *J. Comp. Neurol.* 122 369–383 10.1002/cne.901220307 14184860

[B79] Orlovsky G. N. Deliagina T. G. Grillner S. (1999). *Neuronal Control of Locomotion. From Mollusc to Man.* Oxford: Oxford University Press

[B80] Orlovsky G. N. Deliagina T. G. Wallen P. (1992). Vestibular control of swimming in lamprey. I. Responses of reticulospinal neurons to roll and pitch. *Exp. Brain Res.* 90 479–488 10.1007/BF00230930 1426108

[B81] Pavlova E. L. Deliagina T. G. (2002). Responses of reticulospinal neurons in intact lamprey to pitch. *J. Neurophysiol.* 88 1136–1146 12205135 10.1152/jn.2002.88.3.1136

[B82] Pavlova E. L. Deliagina T. G. (2003). Asymmetry in the pitch control system of the lamprey caused by a unilateral labyrinthectomy. *J. Neurophysiol.* 89 2370–2379 10.1152/jn.00830.2002 12740399

[B83] Petras J. M. (1967). Cortical, tectal and tegmental fiber connections in the spinal cord of the cat. *Brain Res.* 6 275–324 10.1016/0006-8993(67)90196-5 6060511

[B84] Quintern J. Berger W. Dietz V. (1985). Compensatory reactions to gait perturbations in man: short and long term effects of neuronal adaptation. *Neurosci. Lett.* 62 371–376 10.1016/0304-3940(85)90577-4 4094724

[B85] Rushmer D. S. Macpherson J. M. Dunbar D. C. Russell C. J. Windus S. L. (1987). Automatic postural responses in the cat: responses of proximal and distal hindlimb muscles to drop of support from a single hind- or forelimb. *Exp. Brain Res.* 65 527–537 10.1007/BF00235976 3556481

[B86] Schepens B. Stapley P. Drew T. (2008). Neurons in the pontomedullary reticular formation signal posture and movement both as an integrated behavior and independently. *J. Neurophysiol.* 100 2235–2253 10.1152/jn.01381.2007 18632892

[B87] Scinicariello A. P. Eaton K. Inglis J. T. Collins J. J. (2001). Enhancing human balance control with galvanic vestibular stimulation. *Biol. Cybern.* 84 475–480 10.1007/PL00007991 11417059

[B88] Séverac Cauquil A. Martinez P. Ouaknine M. Tardy-Gervet M. F. (2000). Orientation of the body response to galvanic stimulation as a function of the inter-vestibular imbalance. *Exp. Brain Res.* 133 501–505 10.1007/s002210000434 10985684

[B89] Smith P. F. Curthoys I. S. (1989). Mechanisms of recovery following unilateral labyrinthectomy: a review. *Brain Res. Rev.* 14 155–180 10.1016/0165-0173(89)90013-1 2665890

[B90] Stapley P. J. Drew T. (2009). The pontomedullary reticular formation contributes to the compensatory postural responses observed following removal of the support surface in the standing cat. *J. Neurophysiol.* 101 1334–1350 10.1152/jn.91013.2008 19118108

[B91] Strick P. L. Sterling P. (1974). Synaptic termination of afferents from the ventrolateral nucleus of the thalamus in the cat motor cortex. A light and electron microscopy study. *J. Comp. Neurol.* 153 77–106 10.1002/cne.901530107 4817346

[B92] Swadlow H. A. (2002). Thalamocortical control of feed-forward inhibition in awake somatosensory “barrel” cortex. *Philos. Trans. R. Soc. Lond. B Biol. Sci.* 357 1717–1727 10.1098/rstb.2002.1156 12626006 PMC1693091

[B93] Tamarova Z. A. Sirota M. G. Orlovsky G. N. Deliagina T. G. Beloozerova I. N. (2007). Role of GABAA inhibition in modulation of activity of pyramidal tract neurons during postural corrections. *Eur. J. Neurosci.* 25 1484–1491 10.1111/j.1460-9568.2007.05413.x 17425574 PMC2777253

[B94] Ting L. H. (2007). Dimensional reduction in sensorimotor systems: a framework for understanding muscle coordination of posture. *Prog. Brain Res.* 165 299–321 10.1016/S0079-6123(06)65019-X 17925254 PMC4121431

[B95] Toyama K. Tsukahara N. Udo M. (1968). Nature of cerebellar influences upon the red nucleus neurons. *Exp. Brain Res.* 4 292–310 10.1007/BF00235697 5712688

[B96] Ullén F. Deliagina T. G. Orlovsky G. N. Grillner S. (1995a). Spatial orientation of lamprey. 1. Control of pitch and roll. *J. Exp. Biol.* 198 665–673 9318405 10.1242/jeb.198.3.665

[B97] Ullén F. Deliagina T. G. Orlovsky G. N. Grillner S. (1995b). Spatial orientation of lamprey. 2. Visual influence on orientation during locomotion and in the attached state. *J. Exp. Biol.* 198 675–681 9318418 10.1242/jeb.198.3.675

[B98] Ullén F. Deliagina T. G. Orlovsky G. N. Grillner S. (1996). Visual potentiation of verstibular responses in lamprey reticulospinal neurons. *Eur. J. Neurosci.* 8 2298–2307 10.1111/j.1460-9568.1996.tb01193.x 8950094

[B99] White E. L. (1989). *Cortical Circuits.* Boston: Birkhauser

[B100] Yakovenko S. Krouchev N. Drew T. (2011). Sequential activation of motor cortical neurons contributes to intralimb coordination during reaching in the cat by modulating muscle synergies. *J. Neurophysiol.* 105 388–409 10.1152/jn.00469.2010 21068260

[B101] Zelenin P. V. (2011). Reticulospinal neurons controlling forward and backward swimming in the lamprey. *J. Neurophysiol.* 105 1361–1371 10.1152/jn.00887.2010 21248057

[B102] Zelenin P. V. Beloozerova I. N. Sirota M. G. Orlovsky G. N. Deliagina T. G. (2010). Activity of red nucleus neurons in the cat during postural corrections. *J. Neurosci.* 30 14533–14542 10.1523/JNEUROSCI.2991-10.2010 20980611 PMC2988228

[B103] Zelenin P. V. Deliagina T. G. Grillner S. Orlovsky G. N. (2000). Postural control in the lamprey – a study with neuro-mechanical model. *J. Neurophysiol.* 84 2880–2887 11110817 10.1152/jn.2000.84.6.2880

[B104] Zelenin P. V. Grillner S. Orlovsky G. N. Deliagina T. G. (2001). Heterogeneity of the population of command neurons in the lamprey. *J. Neurosci.* 21 7793–7803 11567070 10.1523/JNEUROSCI.21-19-07793.2001 PMC6762887

[B105] Zelenin P. V. Grillner S. Orlovsky G. N. Deliagina T. G. (2003a). The pattern of motor coordination underlying the roll in the lamprey. *J. Exp. Biol.* 206 2557–2566 10.1242/jeb.00451 12819263

[B106] Zelenin P. V. Pavlova E. L. Grillner S. Orlovsky G. N. Deliagina T. G. (2003b). Comparison of the motor effects of individual vestibulo- and reticulospinal neurons on dorsal and ventral myotomes in lamprey. *J. Neurophysiol.* 90 3161–3167 10.1152/jn.00555.2003 12917388

[B107] Zelenin P. V. Hsu L.-J. Orlovsky G. N. Deliagina T. G. (2012). Use of galvanic vestibular feedback to control postural orientation in decerebrate rabbits. *J. Neurophysiol.* 107 3020–3026 10.1152/jn.00042.2012 22402660 PMC3378369

[B108] Zelenin P. V. Lyalka V. F. Hsu L.-J. Orlovsky G. N. Deliagina T. G. (2013). Effects of reversible spinalization on individual spinal neurons. *J. Neurosci.* 33 18987–18998 10.1523/JNEUROSCI.2394-13.2013 24285903 PMC3841459

[B109] Zelenin P. V. Orlovsky G. N. Deliagina T. G. (2007). Sensory-motor transformation by individual command neurons. *J. Neurosci.* 27 1024–1032 10.1523/JNEUROSCI.4925-06.2007 17267556 PMC6673182

